# IL-10 based immunomodulation initiated at birth extends lifespan in a familial mouse model of amyotrophic lateral sclerosis

**DOI:** 10.1038/s41598-020-77564-3

**Published:** 2020-11-30

**Authors:** Michael R. Strickland, Kristen R. Ibanez, Mariya Yaroshenko, Carolina Ceballos Diaz, David R. Borchelt, Paramita Chakrabarty

**Affiliations:** 1 Center for Translational Research in Neurodegenerative Disease, University of Florida, Gainesville, FL 32610 USA; 2 Department of Neuroscience, University of Florida, Gainesville, FL 32610 USA; 3 McKnight Brain Institute, University of Florida, Gainesville, FL 32610 USA; 4 Present Address: Department of Neuroscience, Washington University, St. Louis, MN USA

**Keywords:** Neuroscience, Diseases of the nervous system, Amyotrophic lateral sclerosis

## Abstract

Inflammatory signaling is thought to modulate the neurodegenerative cascade in amyotrophic lateral sclerosis (ALS). We have previously shown that expression of Interleukin-10 (IL-10), a classical anti-inflammatory cytokine, extends lifespan in the SOD1-G93A mouse model of familial ALS. Here we test whether co-expression of the decoy chemokine receptor M3, that can scavenge inflammatory chemokines, augments the efficacy of IL-10. We found that recombinant adeno-associated virus (AAV)-mediated expression of IL-10, alone, or in combination with M3, resulted in modest extension of lifespan relative to control SOD1-G93A cohort. Interestingly neither AAV-M3 alone nor AAV-IL-10 + AAV-M3 extend survival beyond that of the AAV-IL-10 alone cohort. Focused transcriptomic analysis revealed induction of innate immunity and phagocytotic pathways in presymptomatic SOD1-G93A mice expressing IL-10 + M3 or IL-10 alone. Further, while IL-10 expression increased microglial burden, the IL-10 + M3 group showed lower microglial burden, suggesting that M3 can successfully lower microgliosis before disease onset. Our data demonstrates that over-expression of an anti-inflammatory cytokine and a decoy chemokine receptor can modulate inflammatory processes in SOD1-G93A mice, modestly delaying the age to paralysis. This suggests that multiple inflammatory pathways can be targeted simultaneously in neurodegenerative disease and supports consideration of adapting these approaches to treatment of ALS and related disorders.

## Introduction

Innate immune activation is an inherent feature of the neurodegenerative cascade in amyotrophic lateral sclerosis (ALS), a disease characterized by inexorable motor neuron degeneration leading to paralysis and death^1^. Data from patient autopsy as well as rodent models of familial ALS (fALS) clearly demonstrate induction of an inflammatory phenotype in areas of motor neuron injury, leading to the hypothesis that immune activation may play a key role in ALS pathogenesis. This idea is further supported by recent findings that several genetic risk factors for ALS, such as *C9orf72*, *TREM2*, *PROGRANULIN* and *TBK1*, are expressed from microglia, thus reinforcing the idea of a direct pathogenic role of altered immune signaling in ALS^2^.

In the CNS, the major immune cells are microglia and astrocytes—whether activation of these cells indeed trigger neurodegenerative changes, or these cells are incidental actors as bystanders to neuronal injury is still debated. Some of the earliest datasets showing that microglia and astrocytes affect disease progression in fALS mice strengthened the idea that non-cell autonomous signaling from immune cells can promote ALS type neurodegeneration^3–5^. Similarly, *C9orf72* deficient mice have altered myeloid cell function leading to an autoimmune phenotype, suggesting a shared etiologic link between ALS/FTD and immunity^6^. However, given that immune signaling can either have a beneficial or detrimental effect based on the context and environmental cues, therapeutically targeting microglial signaling to effect neuroprotection in ALS has been particularly challenging.

We have previously demonstrated that life-long overexpression of the anti-inflammatory cytokine, murine Interleukin (IL)-10, by recombinant adeno associated virus (AAV) injection in spinal cords of the SOD1-G93A mouse model of fALS significantly increases survival^7^. Although the effect of IL-10 expression in SOD1-G93A mice was modest, we believe that this neonatal delivery paradigm established a useful screening approach for modulating neuroinflammation in ALS models. We also found that IL-10 expression is accompanied by reduced levels of inflammatory chemokines, such as Ccl3 and Ccl5 in the end stage SOD1-G93A mice^7^. Therefore, we hypothesized that the disease modifying mechanism in the IL-10 expressing mice likely involves suppression of the chemokine storm that accompanies end-stage motor neuron injury. This idea is consistent with data from other groups showing that chemokine-driven immune cell activation is a key trigger in ALS pathogenesis and that its attenuation results in disease amelioration^8–10^.

Based on this line of thinking, we wanted to examine whether spinal cord directed inhibition of chemokine signaling will further extend survival in the SOD1-G93A mouse model of fALS. To avoid the complex and redundant signaling pathways inherent in the chemokine system, we decided to use chemokine decoy receptor(s) with broad-spectrum inhibitory function to block chemokine signaling in the SOD1-G93A mice. These chemokine decoy receptors, often secreted by viruses, can bind multiple members of each chemokine superfamily which allow the viruses to neutralize chemokine-dependent immune surveillance by the host. These decoy receptors thus can be repurposed to modulate chronic inflammatory conditions^11^. Here, we have specifically tested whether the broad chemokine-scavenging activity of one such chemokine decoy receptor, murine gamma-2-herpesvirus 68 (MHV-68) encoded M3 protein, can attenuate neuropathology and improve lifespan in the SOD1G93 mice. The M3 chemokine receptor produced by MHV-68 can scavenge a wide range of inflammatory chemokines without binding the homeostatic members of their cognate chemokine families, thus effectively blocking inflammatory chemokine signaling^12–14^. Using recombinant AAV-mediated delivery into neonatal SOD1-G93A mice, we tested the effect of expressing M3, either alone or in combination with IL-10. We found that both the IL-10 alone and IL-10 + M3 cohorts showed extended lifespan compared to control mice. However, in the combinatorial group, addition of M3 produced little added benefit to IL-10 in extending lifespan although M3 expression by itself produced a significant reduction in microgliosis without altering lifespan.

## Results

In a previous study, we explored whether attenuating the inflammatory state could alter disease onset or progression in SOD1-G93A mouse of fALS, by using intraspinal injection of AAV2/1 expressing IL-10 into neonatal SOD1-G93A mice (FvB background)^7^. In this previous study, we observed that IL-10 expression prolonged survival by 15% (*p* < 0.005) relative to mice injected with AAV2/1 expressing EGFP. Focused transcriptomic analysis revealed that IL-10 expression significantly reduces a subset of inflammatory chemokines (Ccl3, Ccl11, Ccl4, Cxcl10) that are normally upregulated in moribund control mice. These findings led us to explore whether dampening inflammatory chemokine signaling could alter SOD1-ALS type pathogenesis in this current study.

To modulate chemokine signaling, here we elected to use a scavenger approach using the MHV-68 virus encoded M3 protein^15^ in the SOD1-G93A mouse model. The M3 protein employs structural mimicry to promiscuously bind chemokines (mouse CCL5, CCL3, CCL2, CX3CL1; human CXCL-8 and XCL1)^14^, thereby sequestering them and preventing chemokine homodimerization and binding to G protein-coupled receptors. Consistent with its role in chemokine scavenging, M3 disrupts chemokine-triggered chemotaxis^16^, limits NF-κB activation and lesion progression in atherosclerosis^17^ and blocks chemokine-mediated insulitis in mouse models^18^. M3 does not bind typical inflammatory cytokines, such as IFN-γ, IL-1β, IL-18 and TNF-α^15^.

We cloned the M3 gene (tagged with V5 sequence at the C-terminus) in pAAV and confirmed expression in primary wild type mouse neuroglial cultures (Supplementary Fig. S1A) and spinal cords of SOD1-G93A mice (Supplementary Fig. S1B). The SOD1-G93A mice used in this study was produced by backcrossing the original mouse strain (B6SJL-Tg(SOD1*G93A) 1Gur/J, obtained from Jackson Laboratories) to B6/C3H F1 mice obtained from Jackson Laboratories. This substrain has a longer life expectancy than the original Gurney mouse (median survival of 170–180 days compared to median survival of 120 days seen in the original Gurney mouse on the B6/SJL background). Neonatal SOD1-G93A litters were injected with a cocktail of 2E10 vector genomes of AAV, each injection containing 1E10 vector genomes of one specific AAV: (1) Control group (EGFP + CTR0; CTR0; Naive), (2) IL-10 group (IL-10 + CTR0), (3) M3 group (M3 + CTR0) and (4) Combination group (IL-10 + M3). Such neonatal injections provide a means to conduct cost-effective and relatively moderate throughput screens to examine the effects of neuroimmune modulation on neurodegenerative phenotypes of mouse models^7,19^. The neonatal litters were randomized for P0 injection of AAVs. These mice were aged till the onset of bilateral hindlimb paralysis combined with a body score index of 2+ and designated as the survival cohort (Table 1). We also collected tissues from pre-symptomatic 3 month old ‘sentinel cohort’ to understand the pathological changes preceding end-stage (Table 1). Since there was no statistical difference in survival between the naïve mice and EGFP + CTR0 (referred to as ‘EGFP’ cohort henceforth) or CTR0 injected mice (Supplementary Fig. S1D), we collapsed these three groups as the Control group for our studies.

**Table 1:** Cohort sizes of SOD1-G93A mice used in the study.

Cohort	Survival cohort		Sentinel cohort	
	M	F	M	F
AAV-CTR0	6	3	0	0
AAV-EGFP	3	5	2	4
Naïve	5	8	8	6
AAV-IL-10	6	9	6	6
AAV-M3	5	8	4	6
AAV-IL-10 + AAV-M3	5	7	4	6

The study was divided into two cohorts: one designated as survival cohort which was analyzed at end-stage (paralysis and body score index of 2+) and the other designated as the sentinel cohort that was analyzed at a pre-symptomatic stage (3 months of age).

**Figure 1:**
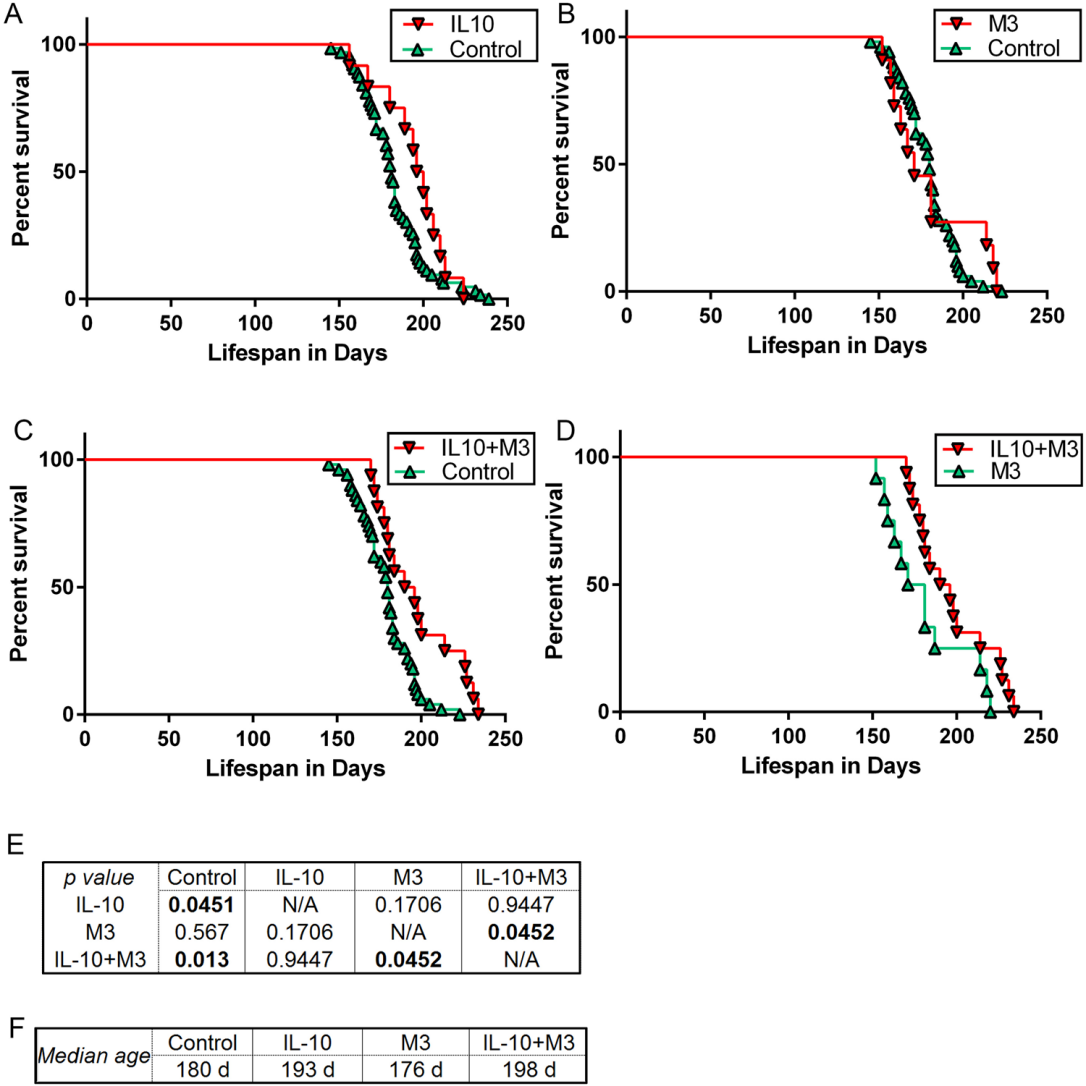
AAV-mediated expression of IL-10 + M3 and IL-10 extends lifespan in SOD1-G93A mice. Kaplan–Meier curves show percent survival of mice over lifespan as measured in days in SOD1-G93A mice aged to paralysis. (**A**) Kaplan–Meier curve of AAV-IL-10 treatment group (median survival 193 days) and control group (Naive, CTR0, and EGFP, median survival 180 days). (**B**) Kaplan–Meier curve of M3 treatment group (median survival 176 days) and control group (median survival 180 days). (**C**,**D**) Kaplan–Meier curve of AAV-IL-10 + AAV-M3 treatment group (median survival 198 days) and control group (median survival 180 days) (**C**) or with M3 group (median survival 176 days) (**D**). Intraspinal injection of AAV-IL-10 + AAV-M3 increased lifespan of mice by 10% relative to control and 12.5% relative to M3. (**E**) Tabulated *p* values (Bonferroni corrected for multiple testing) for each groups of mice derived from Log rank (Mantel–Cox) test. (**F**) Tabulated view of median ages of survival of the different cohorts. n = 30 (Control cohort of AAV-EGFP, AAV-CTR0 and uninjected), 15 (AAV-IL-10), 13 (AAV-M3), 12 (AAV-IL-10 + AAV-M3).

Consistent with our earlier study^7^, we were able to replicate the beneficial effect of IL-10 on SOD1-G93A survival. We found that expression of IL-10 improved survival by 7.2% (*p* = 0.045) in these mice bred on B6/C3H background (Fig. 1A,E,F). In our prior study in the FVB/N strain of G93A mice, IL-10 expression extended survival by 15%^7^. The difference in the magnitude of the effect may be due to the use of different background strains in these studies. The genetic background of mice modulate the ALS phenotype in the SOD1 models and also affects treatment outcomes^20,21^, presumably due to the differential levels of modifier genes in different mouse strains. Though we did not observe any significant changes in survival of SOD1-G93A in the presence of M3 alone (Fig. 1B), the IL-10 + M3 group survived longer compared to the M3 group alone (12.5%; *p* = 0.045) as well as Control cohort (10%; *p* = 0.013) (Fig. 1C–F). There was no significant difference in survival between the IL-10 + M3 and IL-10 group.

**Figure 2:**
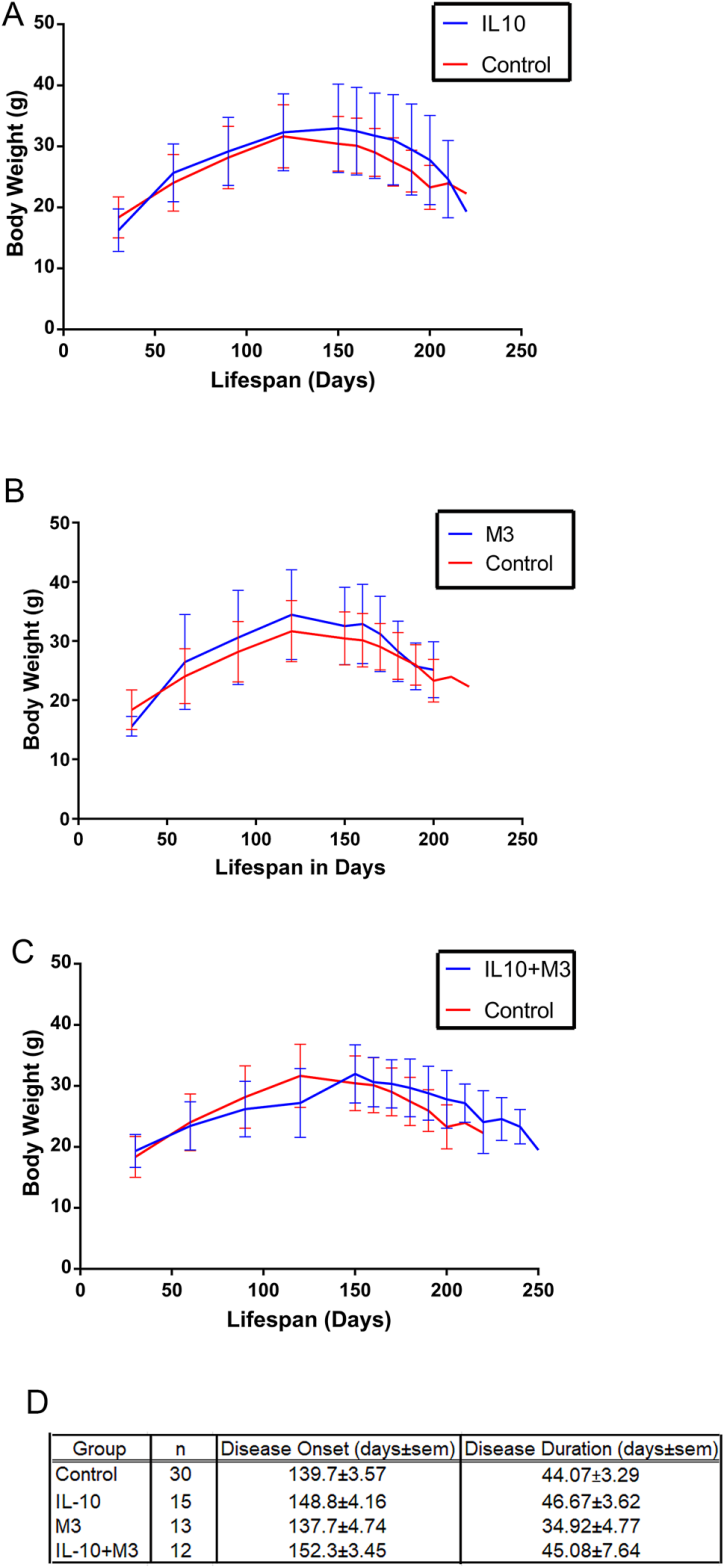
Assessment of disease onset and disease duration in IL-10 and IL-10 + M3 expressing SOD1-G93A mice. Average body weight (mean ± SD) measured in grams over lifespan, in days, of SOD1-G93A mice injected neonatally with different AAVs and aged to end point. (**A**) Body weight in IL-10 treatment group and control group (Naive, CTR0, and EGFP). (**B**) Body weight in grams in M3 treatment group and control group. (**C**) Body weight in grams IL-10 + M3 treatment group and control group. (**D**) Tabulated view of the disease onset age (mean ± sem) and average disease duration (mean ± SEM) representing difference in days between euthanasia due to paralysis and peak body weight. n = 30 (Control cohort of AAV-EGFP, AAV-CTR0 and uninjected combined), 15 (AAV-IL-10), 13 (AAV-M3), 12 (AAV-IL-10 + AAV-M3).

**Figure 3:**
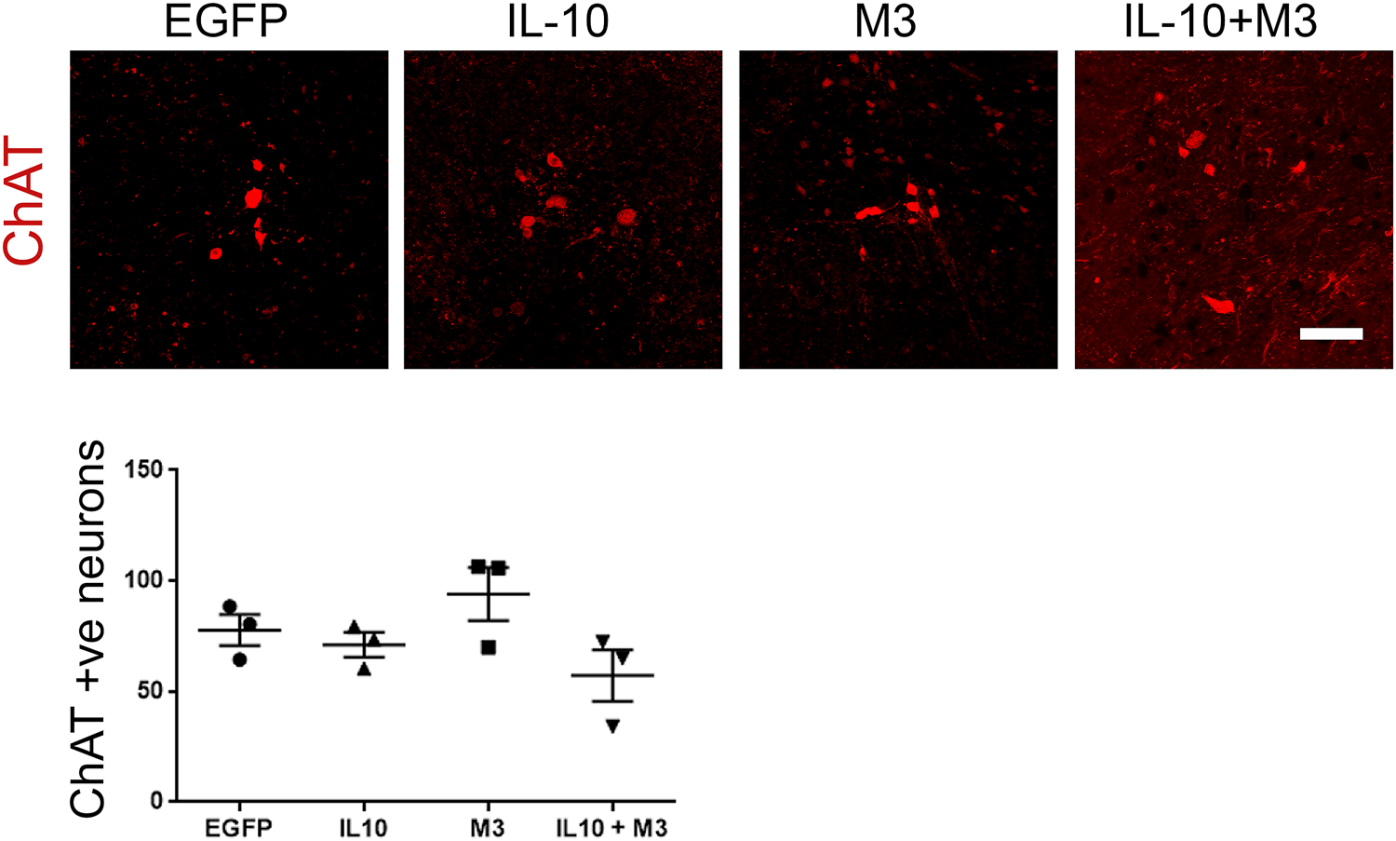
ChAT immunoreactivity in spinal motoneurons is unaltered at end-stage following expression of IL-10 or IL-10 + M3. Averaged intensity of ChAT immunostaining in the cervical-thoracic region, representative of surviving motoneurons, shows no difference in the AAV-IL-10, AAV-M3 or AAV-IL-10 + AAV-M3 expressing mice compared to AAV-EGFP expressing mice in the cervical-thoracic parts of the spinal cord. Motoneurons were stained with anti-ChAT antibody and detected by Alexa fluor 594 nm in a Keyence BZ-X100 microscope. Analysis of cell numbers was done using the BZ-analyzer. N = 3 mice/group.

We used body weight analysis to infer disease onset in these cohorts (Fig. 2). Disease onset was defined as the inflection point in the body weight curve and disease duration was defined as the number of days from this inflection point to the day of euthanasia. Both IL-10 and IL-10 + M3 cohorts showed a non-significant trend towards later disease onset compared to control and M3 cohorts, although the differential onset times were not significant in a group-wise statistics test (Fig. 2A–D). Next we investigated whether there was any difference in the number of surviving ChAT immunopositive motoneurons in end-stage SOD1-G93A mice expressing the anti-inflammatory mediators (Fig. 3). The number of spinal motoneurons in these end-stage mice would serve as a measure of the neurodegenerative process. On averaging the number of motoneurons in the cervical-thoracic segments immuno-stained with choline acetyl transferase (ChAT), we did not observe any significant difference in motoneuron survival across the different groups expressing IL-10 or M3 (Fig. 3).

**Figure 4:**
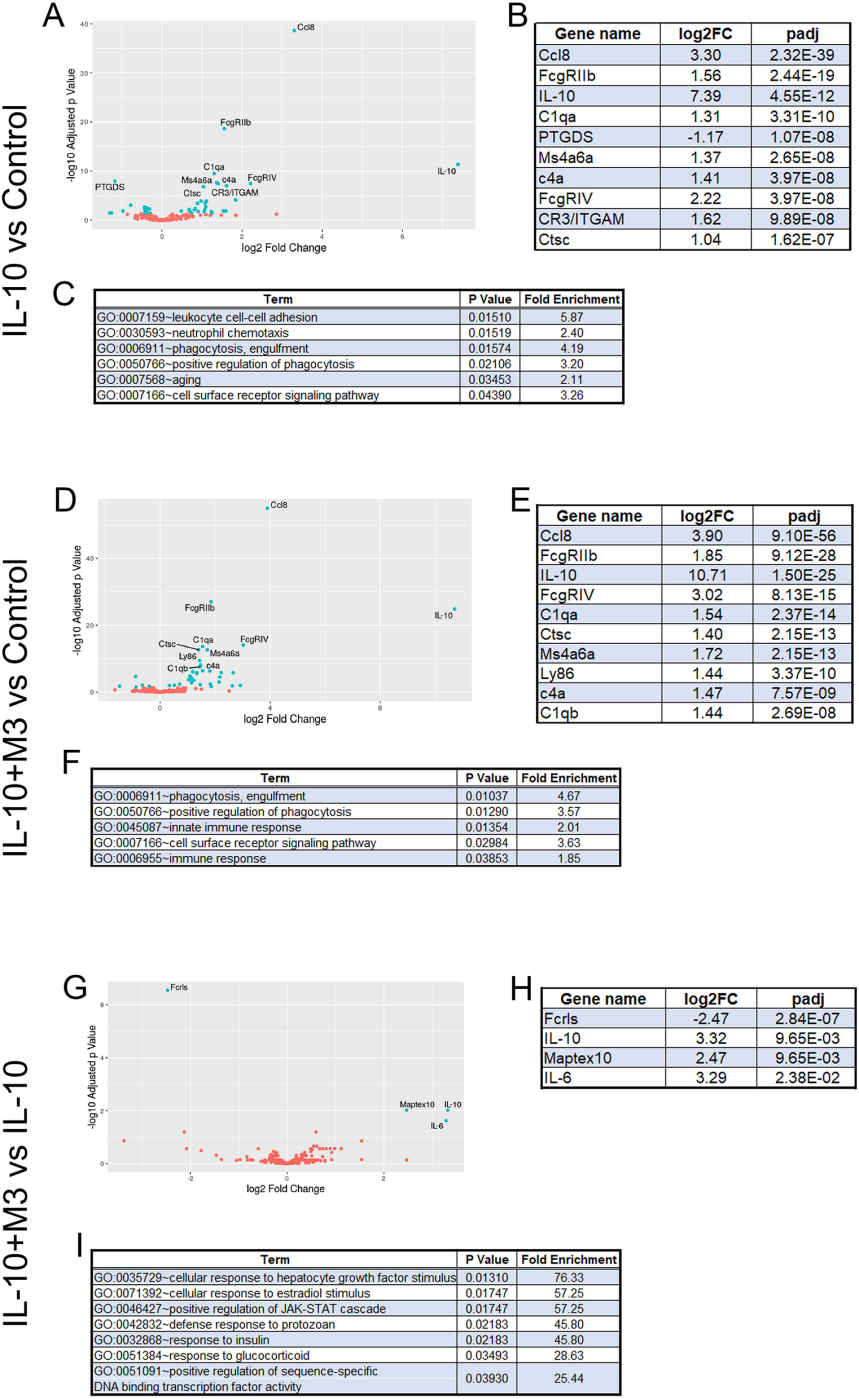
Focused transcriptomic analysis shows differential effects of IL-10 and IL-10 + M3 expression in pre-symptomatic SOD1-G93A mice. A NanoString custom codeset was used to infer transcriptomic changes in AAV-IL-10 (**A**–**C**) and AAV-IL-10 + AAV-M3 (**D**–**F**) relative to AAV-EGFP expressing mice. Mice from each treatment group were submitted for analysis using a custom NanoString array which measured expression level of genes related to inflammation and neurodegeneration. (**A**) Volcano plot of differentially expressed genes in AAV-IL-10 treated relative to AAV-EGFP (control) mice. (**B**) Top 10 most differentially expressed genes in AAV-IL-10 expressing mice relative to control EGFP-expressing mice. (**C**) DAVID pathway analysis of differentially expressed genes in AAV-IL-10 expressing mice compared to control mice. Results show significant upregulation in phagocytosis, aging and cell migration pathways. (**D**) Volcano plot of differentially expressed genes in AAV-IL-10 + AAV-M3 expressing relative to AAV-EGFP (control) mice. (**E**) Top 10 most differentially expressed genes in AAV-IL-10 + M3 treated compared to control mice. (**F**) DAVID pathway analysis of differentially expressed genes in AAV-IL-10 + M3 expressing mice compared to control mice. Upregulated pathways include those involved in phagocytosis and innate immune signaling. (**G**) Volcano plot of differentially expressed genes in AAV-IL-10 + AAV-M3 relative to AAV-IL-10 mice. (**H**) Differentially expressed genes in AAV-IL-10 + AAV-M3 treated mice compared to AAV-IL-10 mice. (**I**) DAVID pathway analysis of differentially expressed genes in AAV-IL-10 + AAV-M3 expressing mice compared to AAV-IL-10 expressing mice. Significantly upregulated pathways includes the cellular response to hepatocyte growth factor. N = 3 mice/group. log2 FC = log2 of fold change; padj = Benjamini–Hochberg adjusted *p* value.

We next used a NanoString custom codeset to conduct a focused transcriptomic analysis of the affected pathways in pre-symptomatic sentinel cohort of SOD1-G93A mice (Fig. 4; Supplementary Fig. S2; Supplementary Tables S1–S3). This custom code-set of 240 genes comprises of key inflammatory and proteostasis mediators relevant to studying neurodegenerative proteinopathy cascades^22^. The PCA analysis and sample to sample distance matrix showed that the IL-10 and IL-10 + M3 groups segregated from the EGFP expressing control SOD1-G93A mice (Supplementary Fig. S2). Among the 240 total genes examined, 43 genes were significantly altered in the IL-10 group whereas 38 genes were altered in the IL-10 + M3 group compared to EGFP expressing cohorts (Supplementary Tables S1–S2). Compared to AAV-EGFP expressing mice, we found that both IL-10 as well as IL-10 + M3 expression resulted in very similar gene expression patterns, notably increased FcgrIIb, Ccl8, Ms4a6a and complement proteins (Fig. 4A,B,D,E). Interestingly, there were several genes that were altered exclusively in each cohort, suggesting that the effect of co-expression of IL-10 and M3 and that of IL-10 alone was distinctive (Table 2). On the whole, gene ontogeny (GO) pathway analysis shows involvement of similar immune signaling pathways in the IL-10 and the IL-10 + M3 cohorts such as positive regulation of phagocytosis and cell surface receptor signaling (Fig. 4C,F). In the IL-10 + M3 cohort, pathways associated with the innate immune response were upregulated whereas in the IL-10 alone cohort, we found pathways associated with leukocyte adhesion and neutrophil chemotaxis were being upregulated (Fig. 4C,F). On comparing the DEG of the IL-10 + M3 to the IL-10 alone cohort, we identified only few genes that reached significance after correcting for multiple testing (Fig. 4G–I; Supplementary Tables S3). One of the genes was IL-10 itself (p(adj) = 0.00965), which would indicate that modulating the inflammatory chemokine pathways via M3 overexpression results in increased IL-10 expression. Since the IL-10 expressed by our AAV was untagged, we cannot determine whether this IL-10 is endogenous or produced from the AAV. We also noted that IL-10 + M3 expression led to increased IL-6 and tau (indicated by Mapt exon 10 sequence), while there was reduction in Fcrls gene expression compared to IL-10 alone cohort (Fig. 4G,H).

**Table 2:** Gene expression in SOD1-G93A mice.

Gene name	log2FC	padj
**Genes uniquely altered in IL-10 cohort**		
Fcrls	1.84	7.62E−05
mSOD1	− 0.43	0.002
Hprt1	− 0.39	0.003
GAPDH	− 0.31	0.005
Pink1	− 0.43	0.007
Mapk8	− 0.38	0.007
hSOD1	− 0.40	0.007
Itgb2/MAC-1	1.05	0.018
Creb1	− 0.42	0.032
eif2alpha	− 0.38	0.033
Ccl11	− 1.26	0.037
CARD9	0.74	0.039
SNCA	− 0.42	0.041
Cfl1	− 0.29	0.042
**Genes uniquely altered in IL-10 + M3 cohort**		
Cd68	1.04	1.98E−04
B2M	1.16	6.69E−04
Cxcl9	2.14	9.00E−04
Cfb	1.46	5.78E−03
Ctsd	0.34	1.07E−02
IL-6	2.92	0.011
MARCO	2.63	0.013
Limk1	− 0.40	0.019
Mapt ex10	1.86	0.021

List of significant DEGs in the AAV-IL-10 cohort and the AAV-IL-10 + AAV-M3 cohort that are exclusively altered in individual cohort. Data is derived from RNA analysis using a NanoString custom code set. log2 FC = log2 of fold change; padj = Benjamini–Hochberg adjusted *p* value.

We next examined the SOD1-G93A mice at two different age groups (pre-symptomatic sentinel and symptomatic end-stage cohorts) to detect alterations in proteinopathy and gliosis burden. The sentinel cohorts were analyzed at 3 months of age to reflect prodromal processes at a pre-symptomatic phase whereas the survival cohort were collected from the paralyzed ~ 6 month old mice to signify end stage processes. Immunoreactivity analysis was also done in two areas of the spinal cord—the gray matter to understand whether changes were originating predominantly in the neuronal cells and in the white matter to assess changes occurring predominantly in the non-neuronal populations. First, we used ubiquitin immunostaining to evaluate the proteinopathy alterations in the SOD1-G93A mice (Supplementary Fig. S3). Based on the fact that protein aggregates in ALS patients and mouse models are characterized by extensive ubiquitination (reviewed in Ref.^23^), we decided to use ubiquitin immunostaining as a surrogate marker for intracellular inclusion formation. Immunohistochemistry with anti-Ubiquitin antibody showed that neither IL-10 or IL-10 + M3 cohorts showed significant differences in the proteinopathy burden in the pre-symptomatic stage or the end-stage compared to the control cohort (Fig. S3A–C).

We next investigated how IL-10 or IL-10 + M3 altered glial burden in the pre-symptomatic and end-stage SOD1-G93A mice. We used the anti-GFAP to detect and quantify astrocytosis in these mice (Supplementary Fig. S4). Overall, there was no significant alterations of the treatment groups relative to the control SOD1-G93A mice in the end-stage cohorts, though there were some subtle changes noted in the sentinel cohort (Supplementary Fig. S4A–C). In both the gray and white matter of pre-symptomatic sentinel mice, we observed that mice expressing IL-10 + M3 had higher levels of astrogliosis markers than mice expressing M3 alone (Supplementary Fig. S4A-C; *p* < 0.05 relative to M3 in gray matter and *p* < 0.05 relative to IL-10 in white matter). At end-stage, there was no detectable difference in GFAP immunoreactivity between any of the cohorts in either the gray matter or the white matter areas (Supplementary Fig. S4D–F).

**Figure 5:**
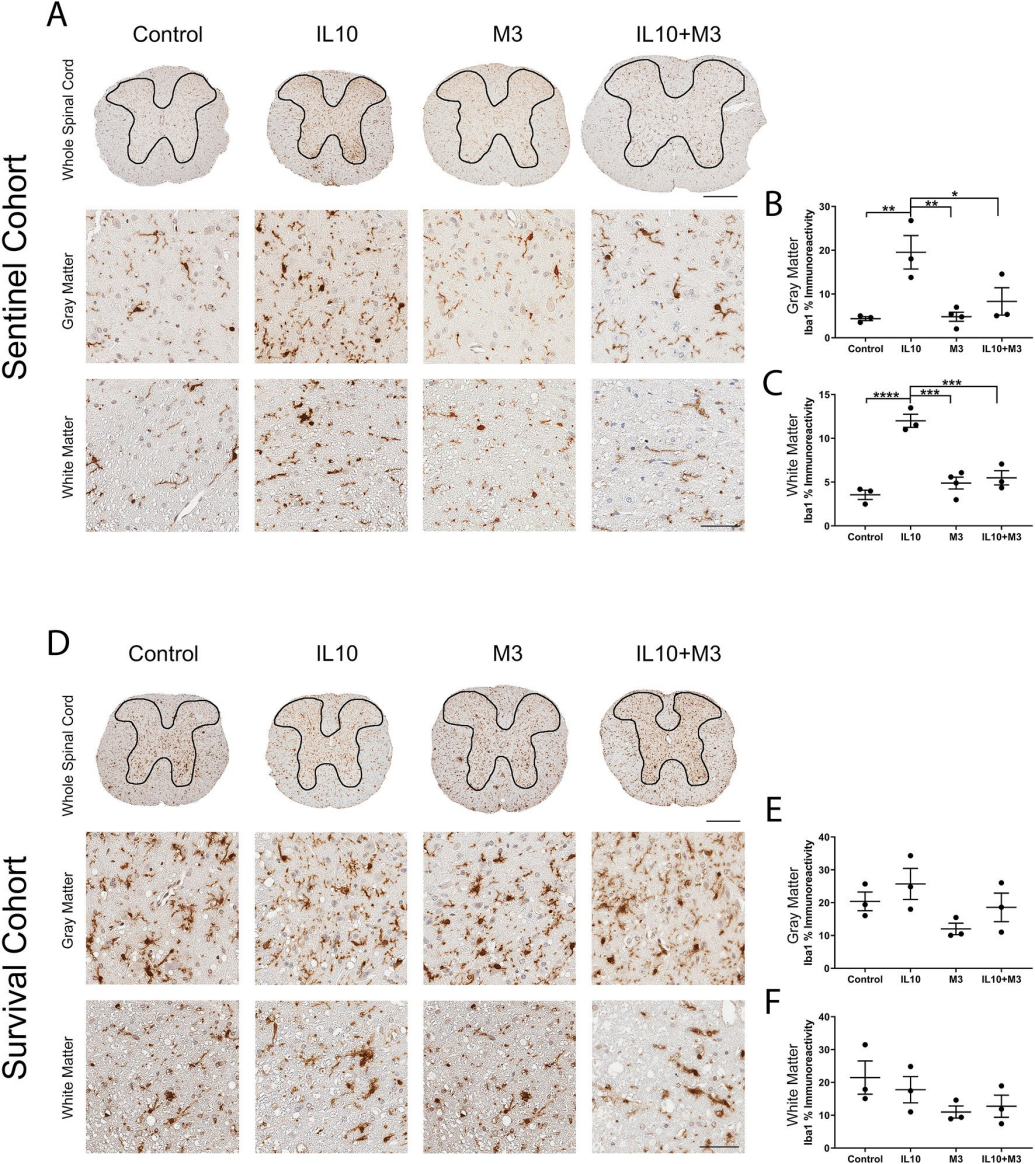
Induction of microgliosis in AAV-IL-10 expressing SOD1-G93A mice in the pre-symptomatic disease stage. Iba-1 antibody was used to examine microgliosis in SOD1-G93A mice neonatally injected with AAV-EGFP, AAV-IL-10, AAV-M3, or AAV-IL-10 + AAV-M3 and aged to 3 months (**A**–**C**) or to end point characterized by bilateral hind limb paralysis (**D**–**F**). Representative images of Iba-1 immunostaining for the whole spinal cord or a magnified view of the gray matter and white matter are shown for the sentinel cohort (**A**) and survival cohort (**D**). The gray matter region is bound within the black outline on the whole spinal cord images. Scale bar, 300 µm (whole spinal cord, top row); scale bar, 50 µm (white matter and gray matter images, bottom rows). The gray matter region of interest (**B**,**E**) outlined in the whole spinal cord images was quantified using the Aperio Positive Pixel Count program, n = 3–4 mice/group. White matter region (**C**,**F**; outside the black outline) was also quantified similarly. Quantification of Iba-1 immunostaining is presented as % immunoreactivity and depicted as mean ± sem. Data was analyzed with One-way ANOVA with Tukey multiple comparisons test. *p < 0.05, **p < 0.01, ***p < 0.005, ****p < 0.001. Quantification showed upregulation of microgliosis of SOD1-G93A mice injected with AAV-IL-10 compared to other groups in both white matter and gray matter regions (**B**,**C**). No significant changes in immunoreactivity were observed in white matter or gray matter regions in end stage mice (**E**,**F**).

Using Iba-1 immunoreactivity as an indicator of microglial burden, we observed that IL-10 expression led to increased microgliosis in both the gray matter and white matter of the sentinel mice compared to control mice (Fig. 5A–C; *p* < 0.01 in grey matter, *p* < 0.0001 in white matter). The IL-10 + M3 sentinels, in spite of expressing higher amounts of IL-10 (Fig. 4G), surprisingly showed lower microglial burden relative to the IL-10 mice (Fig. 5A–C; *p* < 0.05 in gray matter, *p* < 0.001 in white matter). In end-stage mice, we did not observe any statistically significant changes in microgliosis in between the experimental groups (Fig. 5D–F). Taken together, this suggests that M3 can temper the proliferative effects of IL-10 on spinal cord microglia in prodromal stages.

## Discussion

The underlying pathophysiology of ALS is not fully understood, though several groups including ours have shown that neuroinflammation, as exemplified by increased inflammatory cytokine and chemokine expression, can result in neuronal injury and disease progression^1^. In ALS patients, higher plasma IL-10 levels correlated with longer disease duration, suggesting some ameliorative effects of innate immune homeostasis^24^. Consistent with this, we have shown previously that intraspinal expression of the anti-inflammatory cytokine, IL-10, extends lifespan in SOD1-G93A model^7^. Following this, another group confirmed that microglial IL-10 overexpression delayed disease and antibody-mediated IL-10 blockade accelerated disease in fALS mice^25^. Based on the fact that inflammatory chemokines play a deterministic role in ALS progression in mice^8^ and humans^26^, we reasoned that a combinatorial therapy consisting of IL-10 and an anti-chemokine agent could have an additive synergistic positive effect in disease onset and progression by targeting multiple neuroinflammatory pathways in the CNS of SOD1-G93A mice. As established in our previous study showing that AAV could be used to deliver secreted immune modulators to be effective in a non cell autonomous fashion^7^, we used intraspinal injection of AAV1 encoding IL-10 or chemokine scavenger M3 in neonatal SOD1-G93A mice to modulate inflammatory signaling. Here we show that (1) combinatorial administration of AAV-IL-10 and AAV-M3 in neonatal mice leads to increased lifespan and delayed disease onset in SOD1-G93A mice relative to control cohorts, although the expression of M3 does not extend the efficacy beyond that of IL-10 alone and (2) expression of M3 in the combinatorial setting reduces the propensity of IL-10 induced microgliosis in the presymptomatic phase. Although we observed that the expression of M3 attenuated microgliosis caused by expression of IL-10, the combinatorial administration of AAV-IL-10 and AAV-M3 produced no significant benefit over IL-10 alone. Our findings show that combinatorial expression of immune modulators is feasible using AAV, and that additional work is required to identify factors that can more strongly modulate inflammatory processes in the SOD1-G93A model of ALS.

Chemokines, involved in leukocyte chemoattraction and immune response, have been shown to be involved in disease progression in multiple systemic diseases as well as in neurodegenerative diseases such as ALS^8^. Though neutralizing chemokine signaling to achieve therapeutic benefits is attractive, it is challenging to do so because of the high redundancy of the chemokine system. On the other hand, atypical chemokine receptors that have decoy and/or scavenger functions can effectively target multiple chemokine sub-families simultaneously and thus generally block diverse chemokine signaling pathways^27,28^. Several such chemokine binding proteins are naturally produced by viruses and even some higher organisms as a means to effectively modulate chemokine-mediated immune response^27^.

In our study, we used the M3 protein, a chemokine binding protein produced by the MHV68 virus, which is functionally unique as this is probably the only known chemokine binding-protein that binds and scavenges all four chemokine subfamily members with high affinity^15,29^. The M3 decoy receptor is also considered to be a ‘silent’ scavenger as it does not induce Ca^2+^ flux or chemotaxis by untimely release of its sequestered cargo^30^. It is a secreted glycoprotein and has been used in several preclinical models to successfully modulate immunity and therapeutically alleviate immunopathology^13,31,32^. Because of its soluble nature and its previously demonstrated positive therapeutic effect in a mouse model of experimental autoimmune encephalitis where it reduced neuroinflammation^33^, we hypothesized that M3 might be effective as another arm of anti-inflammatory strategies to delay ALS progression. Consistent with this idea, we found that M3 expression attenuated microglial proliferation induced by IL-10 over-expression but was unable to cause disease modification in SOD1-G93A mice.

Using a focused transcriptomic array, we found that DEG of IL-10 and IL-10 + M3 mice were mostly similar, though we found significantly increased levels of IL-10 and IL-6 in the latter group. The significant increase in IL-10 in the IL-10 + M3 group relative to the IL-10 group, even after controlling for viral loads administered and ensuring both groups were injected with two AAVs of equivalent titer, opens up interesting issues. Though we cannot discount that co-injecting the two AAVs may influence each other, this seems unlikely as several groups have co-injected AAVs of same serotype without observing any change in infectivity, transduction or expression characteristics^34,35^. Alternatively, our data would suggest that M3 dependent signaling pathways can increase endogenous or recombinant AAV-derived IL-10 production. The transcriptional mechanisms regulating IL-10 expression from different cells is extremely complex and whether downregulation of specific inflammatory chemokines is one such mechanism remains to be elucidated. Thus, on a facile level, we could infer that simply increasing IL-10 is enough to drive disease modification in SOD1-G93A mice as seen in the IL-10 + M3 injected group. However, we noticed additional subtle but significant differences between IL-10 and IL-10 + M3 groups that would belie such a simple explanation. For example, in the sentinel group of asymptomatic mice, we found that while IL-10 expression increased microglial burden, co-expressing IL-10 + M3 did not have this effect in spite of higher RNA levels of IL-10 in the co-expressing group. This difference is also exemplified in astrogliosis which shows highest astroglial burden in the pre-symptomatic IL-10 + M3 group relative to both IL-10 or M3 groups. We also noticed several genes that were uniquely altered only in the IL-10 group or only in the IL-10 + M3 group relative to controls. Together with the data that microglial response in the IL-10 + M3 mice were similar to M3 mice rather than to IL-10 mice in the pre-symptomatic disease stage suggests that simply driving up IL-10 levels could not have been the primary driver for disease modification in the IL-10 + M3 mice.

We noticed reduced levels of Fcrls in the IL-10 + M3 mice relative to IL-10 mice in our RNA analysis data. In a previous study, Fcrls was identified as part of a unique microglial signature in mice that is lost in mice lacking TGFβ1, showing that reduction in Fcrls is consistent with microglia deficiency^36^. This is also supported by our immunohistochemical data showing reduced microglial burden in the pre-symptomatic IL-10 + M3 mice relative to IL-10 mice. Interestingly, M3 expression by itself did not alter disease trajectory even with lower microglial burden at end-stage compared to IL-10 alone group. Taken together, this would suggest that M3 induced pathways can temper the IL-10 induced microglial proliferation and simultaneously allow IL-10 mediated disease modification in the SOD1-G93A mice. Future studies using systems biology approaches are necessary to mechanistically characterize the microglial and astrocytic phenotypes that can underlie disease modification in such models of neurodegenerative diseases.

Our disease modifying study uses the G93A-SOD1 overexpression mouse model to mimic ALS features. Unfortunately, a number of promising therapies identified using this model could not be successfully translated to humans. Some possible explanations for such poor translation is the high levels of SOD1 overexpression in this model as well as copy number variations within this line^37^. While our litter randomization strategy was designed to overcome the latter issue, we cannot completely rule out that our paradigm is only effective in SOD1 G93A-specific genetic environment. However, as multiple studies have demonstrated that inflammatory pathways are involved in neurodegeneration in both sporadic and familial ALS^6,38^, we believe that anti-inflammatory approaches described in this study has the potential to be effective beyond mutant SOD1-ALS related pathologies.

In conclusion, here we show that life-long expression of the chemokine scavenger M3 moderates microgliosis, but does not appreciably synergize with IL-10 to mediate disease modification in SOD1-G93A mice. Though gene therapy mediated therapies have been successfully used in pediatric patients as preventive therapy^39^, a more translational strategy would be to use direct spinal or intramuscular infusion of pure biologics in symptomatic individuals. Future studies using combinatorial infusions of these biologics (Il-10 in combination with scavenger receptors) into various ALS rodent models at different stages of the disease will need to be done to establish the potential translatability of such anti-inflammatory therapeutic combinations in ALS and related disorders.

## Methods

### AAV construction and preparation

AAV vectors expressing IL-10, M3 or Enhanced Green Fluorescent Protein (EGFP) under the cytomegalovirus enhancer/chicken β-actin promotor (CMV/CBA) and an empty vector with no recombinant insert (CTR0) were packaged in AAV capsid 1 as described earlier^7^. The original M3 plasmid was a kind gift of Dr. Stacey Efstathiou at the University of Cambridge. The genomic titer of each virus was determined by quantitative PCR. Freshly prepared AAVs were aliquoted and stored at − 80 °C. When needed, viruses were diluted in sterile 1X PBS, pH 7.2 and immediately used.

### Mice and neonatal injections

All animal husbandry procedures performed were approved by the University of Florida Institutional Animal Care and Use Committee (IACUC) and followed the University of Florida Animal Care Services (ACS) recommendations. We used the SOD1-G93A model for our experiments; this mouse model develops motor neuron degeneration and muscle weakness leading to hindlimb paralysis, recapitulating the clinical symptoms of human familial and sporadic ALS and is thus considered a gold standard for experimental manipulations. The original SOD1-G93A mouse strain (B6SJL-Tg (SOD1*G93A)1Gur/J, obtained from Jackson Laboratories) was backcrossed to B6/C3H (Harlan) background for 10 generations^40^. This resulting substrain of SOD1-G93A mice develops paralysis around 6 months of age^40^ as opposed to the mice on FvB background, used in our previous study on IL-10, that develops paralysis by 3–4 months of age^7^. Rodents were housed in a specific-pathogen free facility in the University of Florida and underwent normal housing. All animals were housed three to five per cage, maintained on ad libitum food and water, and experienced a 12 h light–dark cycle.

Litters from each breeding pair were assigned to the injection groups on a rotating basis (e.g. if Litter 1 from Breeding Pair 1 is injected with EGFP then the second litter, Litter 2 from Breeding Pair 1, is injected with CTR0 or an experimental variable, and so on). Each mouse spinal cord received 2 × 10^10^ vector genomes (volume of 2 µl) on neonatal day P0 as described earlier^7^. The sample size for transgenic mice used in experiment were 13, 9, and 8 for the control groups consisting of naïve (non-injected), AAV-CTR0, and AAV-EGFP injected mice respectively and 15, 13 and 12 for AAV-IL-10, AAV-M3 and AAV-IL-10 + AAV-M3 respectively (Table 1). The viral load in single AAV injections were normalized to the mice receiving two different AAVs (AAV-IL-10 + AAV-M3) by adding equivalent amounts of AAV-CTR0 to either AAV-IL-10, AAV-M3 or AAV-EGFP. Henceforth, the AAV-IL-10 + AAV-M3 cohort is abbreviated as ‘IL-10 + M3’, AAV-IL-10 + AAV-CTR0 as ‘IL-10’, AAV-M3 + AAV-CTR0 as ‘M3’ and AAV-EGFP + AAV-CTR0 as ‘EGFP’. Mice in the survival cohort were monitored daily and weighed monthly until 5 months of age then weighed every other day. Disease onset was defined as the inflection point in the body weight curve and disease duration was defined as the number of days from this inflection point to the day of euthanasia when these reached endpoint criteria. Criteria for endpoint were determined by complete hind limb paralysis and a body score index (IACUC parameter) of 2+. At endpoint, mice were euthanized by anesthesia overdose and transcardial perfusion with saline. The end-users were blinded to the identity of the mice during downstream neuropathological analysis. AAV injections were done by an independent experimenter unfamiliar with the breeding and euthanasia procedures. Mice that had to be euthanized for any reason other than complete hindlimb paralysis were not considered for data analysis. Kaplan–Meier survival curve analysis (Mantel–Cox test) was done using GraphPad Prism 7.1 software. We followed the essential study criteria established previously to minimize inherent noise in the SOD1-G93A mouse model and allow for true treatment efficacy to be measured^37^.

### Immunohistochemical staining, imaging, and image analysis

Spinal cords were harvested following transcardial perfusion with PBS. Dissected tissues were either immersion fixed overnight in formalin solution at 4 °C (cervical-thoracic part) or flash frozen (lumbar part). Spinal cords were cross-sectionally dissected prior to embedding. The cervical-thoracic portion of the spinal cords were dissected approximately at 3 locations approximately 1 cm apart. 4 parts of the spinal cord, thus obtained, were embedded on the same paraffin blocks representing the whole spinal cord. Paraffin embedded spinal cord sections were stained with ChAT (1:250; # AB144P, Abcam) and Iba-1 (1:1000; # 019-19741, Wako) after antigen retrieval using sodium citrate buffer (10 mM sodium citrate, 0.05% Tween 20, pH 6.0). Spinal cord sections were stained with GFAP (1:4000; # Z0334, Dako) and Ubiquitin (1:375; # 3936S, Cell Signaling) following steam antigen retrieval. Briefly, slides are first deparaffinized, steamed in antigen retrieval solution, blocked in 2% fetal bovine serum (FBS) prepared in 1× PBS for 1 h and incubated with primary antibody overnight. Signal was detected using HRP-labeled secondary antibodies (Vector ImmPRESS) and ImmPACT DAB. Slides were scanned using the Scanscope XT image scanner (Aperio Scanner, Leica Biosystems, Buffalo Grove, IL) and immunohistochemical burden analyzed using pixel count algorithm (Aperio ImageScope v11.1.2.760). Statistical tests were done using 1-Way ANOVA with Tukey multiple comparisons test using GraphPad Prism 7.1 software.

### Calculation of ChAT neurons numbers

Following harvesting, cervical-thoracic portion of the spinal cords were dissected into 4 parts of equal length and were embedded on the same paraffin blocks representing the whole spinal cord. 3 consecutive sections from each mice, spaced 25 µm apart, were used for staining with ChAT antibody and detection with anti goat Alexa Fluor-594 nm secondary antibody slides. Slides were counterstained and mounted with 4′,6-diamidino-2-phenylindole (DAPI; Southern Biotech). Whole slide images were obtained at the same brightness/contrast in the Keyence BZ-X700 (Keyence Corporation of America, Itasca, IL, USA) utilizing the optical sectioning mode and capturing Z-stacks over 20 µm at recommended step-sizes and projected onto a full focus image using the BZ-analyzer. The analyzer was allowed to count single ‘events’ in the red fluorescence channel with a diameter > 10 µm, followed by a second blinded observer testing the sensitivity and specificity of event-calling. Statistical tests were done using Tukey’s multiple comparisons test using GraphPad Prism 7.1 software. Data presented is the mean ± sem value of such events per spinal cord per cohort. All analysis was done in an automated mode using the VH Analyzer software (Keyence). We selected the sample number based on post hoc binomial endpoint method, α = 0.05, power of 95% and an expected difference of 15% reduction between two samples (https://clincalc.com/Stats/Power.aspx).

### Tissue fractionation, protein extraction and immunoblotting

The lumbar-sacral portion of the spinal cord (approximately 1 cm from the distal end) was flash frozen and cryopulverized in the presence of liquid nitrogen and divided equally into two aliquots for RNA extraction or protein extraction. Protein extraction was done by homogenization in RIPA buffer containing protease and phosphatase inhibitors (Pierce Protease and Phosphatase Inhibitor Mini Tablets, ThermoScientific). 30 µg of spinal cord lysate was separated in a 4–20% Tris–glycine gel (Novex, Invitrogen), proteins transferred to PVDF membranes and blocked in 0.5% casein. Membranes were incubated in appropriate primary antibodies (anti V5, 1:1000, ThermoFisher # R960; GAPDH, 1:1000, Abcam # ab181602) and secondary antibodies conjugated to IRDye (1:20,000; Li-Cor Biotechnology) diluted in 0.5% casein and protein bands were detected using the multiplex Li-Cor Odyssey Infrared Imaging system (Li-Cor Biotechnology, Lincoln, NE, USA).

### RNA preparation, NanoString assay and differential gene expression analysis

Mice from the sentinel cohort (harvested ~ 90 days) were selected for RNA analysis and differential gene expression quantification using the NanoString nCounter technology (NanoString Biotechnology, Seattle)^41^. The lumbar-sacral portion of the spinal cord (approximately 1 cm from the distal end) was flash frozen and cryopulverized in the presence of liquid nitrogen for RNA extraction. RNA extraction was done as described earlier and analyzed using NanoString custom panel^22^. The advantage of this technology is that it uses color-coded fluorescent probes to quantify mRNA transcripts avoiding artifacts from sequence amplification. Three mice (2 females, 1 male) from each of the AAV-EGFP (Control), AAV-IL-10, and AAV-IL-10 + AAV-M3 groups were selected for analysis. The count matrix obtained from NanoString was processed using the DESeq2 software package^42^. The count matrix data was imported into the DESeq2 package with the experimental design parameter as the treatment (AAV-EGFP, AAV-IL-10, and AAV-IL-10 + AAV-M3). Differential gene expression analysis was run comparing the AAV-IL-10 to AAV-EGFP, AAV-IL-10 + AAV-M3 to AAV-EGFP, and AAV-IL-10 + AAV-M3 to AAV-IL-10 using the DESeq2 package. DAVID pathway analysis was done comparing statistically significant differentially expressed genes (*p*-adjusted < 0.05) with the background set to the complete NanoString gene list^43^. Volcano plots for the data were generated using ggplot2 based on code provided by the Harvard Chan Bioinformatics Core (Harvard Chan Bioinformatics Core (HBC) 2019; Differential gene expression workshop. Retrieved from https://github.com/hbctraining/DGE_workshop;^44^). Heatmap, sample-to-sample distance matrix, and PCA plot were generated using DESeq2 software package^42^.

### Primary neuronal culture

B6/C3H neonatal pups were used to generate mouse primary neuronal cultures as described before^45^. Cultures plated on 8-well chamber slides (Nunc Lab-Tek CCII Chamber slides, Thermo Scientific) or poly-D-Lysine (Sigma, MO) coated 6 well plates were transduced with 1 × 10^10^ viral genomes of AAV-M3 on day 5 and cells were harvested on day 10 for immunostaining and immunoblotting.

### Statistics

All data presented is mean ± SEM, unless otherwise specified. All multiple comparisons use 1-way Anova with Tukey’s correction. Survival analysis is presented as a Kaplan–Meier analysis using the Mantel–Cox test. All statistics were performed using GraphPad Prism 7.1 software. All western blot and microscope images were captured using same parameters and, if altered, this was done using exactly same parameters in Adobe Photoshop CC 2019.

### Ethics declarations

All rodent experiments were approved by the University of Florida IACUC.

### Consent to publish

Not applicable.

## Supplementary information

Supplementary Legends.

Supplementary Information.

## Data Availability

All data generated or analyzed during this study are included within the article and its supplementary additional files.

## Abbreviations

AAV: Adeno-associated virus
ALS: Amyotrophic lateral sclerosis
ChAT: Choline acetyl transferase
CNS: Central nervous system
CTR0: AAV plasmid backbone with no recombinant construct
EGFP: Enhanced green fluorescent protein
fALS: Familial ALS
FTD: Frontotemporal dementia
GFAP: Glial fibrillary acidic protein
GO: Gene ontogeny
Iba-1: Ionized calcium binding adaptor molecule 1
IL-10: Interleukin-10
p(adj) or padj: *P* value adjusted for multiple testing
PCA: Principle component analysis
SOD1-G93A: Transgenic mice expressing G93A variant of human SOD1
