# Author Correction: IL-10 based immunomodulation initiated at birth extends lifespan in a familial mouse model of amyotrophic lateral sclerosis

**DOI:** 10.1038/s41598-021-83092-5

**Published:** 2021-02-03

**Authors:** Michael R. Strickland, Kristen R. Ibanez, Mariya Yaroshenko, Carolina Ceballos Diaz, David R. Borchelt, Paramita Chakrabarty

**Affiliations:** 1 Center for Translational Research in Neurodegenerative Disease, University of Florida, Gainesville, FL 32610 USA; 2 Department of Neuroscience, University of Florida, Gainesville, FL 32610 USA; 3 McKnight Brain Institute, University of Florida, Gainesville, FL 32610 USA; 4 Present Address: Department of Neuroscience, Washington University, St. Louis, MN USA

Correction to: *Scientific Reports* 10.1038/s41598-020-77564-3, published online 30 November 2020

This Article contains an error in Figure 5, where the image in panel D under the M3—White Matter group is a duplication of the image in panel D under the Control—White Matter group. The correct Figure 5 appears below as Figure 1.

**Figure 1:**
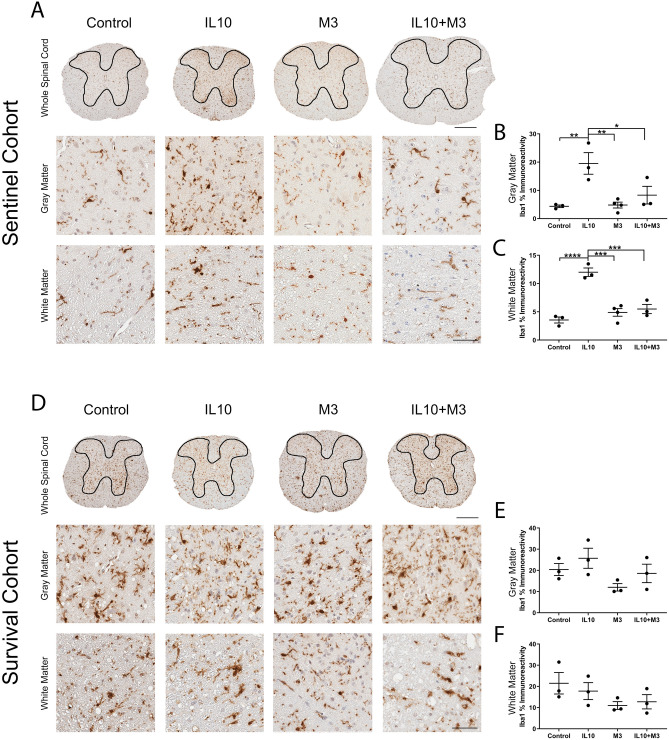
A correct version of the original Figure 5.

