# Supplementary material for: IL-10 based immunomodulation initiated at birth extends lifespan in a familial mouse model of amyotrophic lateral sclerosis
Source: Sci Rep. 2020 Nov 30;10:20862. doi: 10.1038/s41598-020-77564-3 (PMC7705692; doi:10.1038/s41598-020-77564-3)
Supplement: Supplementary file 1 — Supplementary Legends. [file 41598_2020_77564_MOESM1_ESM.docx]

**ADDITIONAL FILES: SUPPLEMENTARY FIGURE LEGENDS AND TABLE LEGENDS.**

**Supplementary Figure S1. Validation of AAV-M3 expression and aggregated survival curves of control SOD1-G93A mice.**

(A-B) AAV-M3 was used to transduce primary neuron cultures from wild type B6/C3H mice on day 5. Immunostaining (A) shows M3 overexpression using anti V5 antibody. MAP2 (detected with Alexa fluor 594 nm) is used as a neuronal marker, V5 (detected with Alexa fluor 488nm) is used to detect M3 whereas DAPI denotes nucleus in the immunostained panel. (B-C) Neonatal SOD1-G93A mice were injected with AAV-EGFP, AAV-M3, or AAV-IL-10+AAV-M3. Immunoblotting of RIPA solubilized cell extracts from the lumbar spinal cord was done and M3 protein detected using anti V5 antibody. GAPDH is used as the loading control. (C) The full length blot for GAPDH from B panel is shown. (D) Survival (Kaplan-Meier) curves of naïve SOD1-G93A mice or SOD1-G93A mice injected intraspinally with AAV-CTR0 or AAV-EGFP is shown. The median survival (in days) is tabulated below the graph. There is no statistical difference between the three groups following Log-rank test. n=13 (naïve), 9 (AAV-CTR0) and 8 (AAV-EGFP).

**Supplementary Figure S2. Principle component analysis (PCA), sample-to-sample distance matrix and heat map of RNA analysis data from SOD1-G93A mice.**

(A) PCA shows separation between the AAV-EGFP treated mice and AAV-IL-10 and AAV-IL-10+AAV-M3 treated mice. (B) The sample-to-sample distance matrix also supports the separation between the treatment and control group as it shows higher correlation between the treatment samples compared to the control samples. (C) Heat map analysis showing differentially expressed genes in SOD1-G93A mice separated by treatment. N=3 mice/group. Sex is indicated as M, male and F, female in each panel.

**Supplementary Figure S3. Assessment of ubiquitin immunoreactivity burden in SOD1-G93A mice.**

Neonatal SOD1-G93A mice were injected with AAV-EGFP, AAV-IL-10, AAV-M3, or AAV-IL-10+AAV-M3 and aged to 3 months (A-C) or to end point characterized by bilateral hind limb paralysis (D-F). Representative images of ubiquitin immunostaining for the whole spinal cord or magnified areas of the gray matter and white matter are shown for the sentinel cohort (A) and survival cohort (D). The gray matter region is bound within the black outline on the whole spinal cord images. Scale bar, 300µm (whole spinal cord, top row); scale bar, 50µm (white matter and gray matter images, bottom rows). The gray matter region of interest (B, E) outlined in the whole spinal cord images was quantified using the Aperio Positive Pixel Count program, n=3-4 mice/group. White matter region (C, F; outside the black outline) was also quantified similarly. Quantification of ubiquitin immunostaining is presented as % immunoreactivity and depicted as mean ± sem. Data was analyzed with 1-way ANOVA with Tukey multiple comparisons test. No significant changes in ubiquitin immunoreactivity were observed in white matter or gray matter regions in either cohort (B, C, E, F).

**Supplementary Figure S4. Assessment of astrogliosis burden in SOD1-G93A mice.**

GFAP antibody was used to examine astrogliosis in neonatal SOD1-G93A mice injected with AAV-EGFP, AAV-IL-10, AAV-M3, or AAV-IL-10+AAV-M3 and aged to 3 months (A-C) or to end point characterized by hind limb paralysis (D-F). Representative images of GFAP positive immunostaining for the whole spinal cord or different regions of the gray matter and white matter are shown for the sentinel cohort (A) and survival cohort (D). The gray matter region is indicated by the black outline on the whole spinal cord images. Scale bar, 300µm (whole spinal cord, top row); scale bar, 50µm (white matter and gray matter images, bottom rows). The gray matter region of interest (B, E) outlined in the whole spinal cord images was quantified using the Aperio Positive Pixel Count program, n=3-4 mice/group. White matter region (C, F; outside the black outline) was also quantified similarly. Quantification of GFAP immunostaining is presented as % immunoreactivity and depicted as mean ± sem. Data was analyzed with One-way ANOVA with Tukey multiple comparisons. *p<0.05. In pre-symptomatic mice, increased GFAP was observed in gray matter region (B) and in white matter region (C) of AAV-IL-10+AAV-M3 injected group relative to AAV-M3 and AAV-IL-10 respectively. No significant changes in immunoreactivity were observed in white matter or gray matter regions in end stage SOD1-G93A mice (E, F).

**Additional File 5: Supplementary Tables.**

**Table S1:** Differential Expression of Genes in AAV-IL-10 vs AAV-EGFP expressing SOD1-G93A mice.

**Table S2:** Differential Expression of Genes in AAV-IL-10+AAV-M3 vs AAV-EGFP expressing SOD1-G93A mice

**Table S3:** Differential Expression of Genes in AAV-IL-10+AAV-M3 vs AAV-IL-10 expressing SOD1-G93A mice
