# Supplementary material for: IL-10 based immunomodulation initiated at birth extends lifespan in a familial mouse model of amyotrophic lateral sclerosis
Source: Sci Rep. 2020 Nov 30;10:20862. doi: 10.1038/s41598-020-77564-3 (PMC7705692; doi:10.1038/s41598-020-77564-3)
Supplement: Supplementary file 2 — Supplementary Information. [file 41598_2020_77564_MOESM2_ESM.pdf]

IL-10 based immunomodulation initiated at birth extends lifespan in a familial mouse model of amyotrophic lateral sclerosis

Michael R Strickland[1,*], Kristen R Ibanez[1], Mariya Yaroshenko[1], Carolina Ceballos Diaz[1], David R Borchelt[1,2,3], Paramita Chakrabarty[1,2,3,#]

[1]Center for Translational Research in Neurodegenerative Disease, University of Florida, Gainesville, FL 32610, USA.

[2]Department of Neuroscience, University of Florida, Gainesville, FL 32610, USA.

[3]McKnight Brain Institute, University of Florida, Gainesville, FL 32610, USA.

*Present Address: Department of Neuroscience, Washington University, St. Louis, MN.

Email Addresses:

Michael R Strickland	mrstrickland@wustl.edu
Kristen Ibanez	kibanez@ufl.edu
Mariya Yaroshenko	mashayaroshenko@gmail.com
Carolina Ceballos Diaz	carocebdi@gmail.com
David R Borchelt	drb1@ufl.edu
Paramita Chakrabarty	pchakrabarty@ufl.edu

#Address correspondence to Paramita Chakrabarty

Email: pchakrabarty@ufl.edu

Phone: 1-352-273-7271

Fax: 1-352-294-5060

Running Title. Immunomodulation in SOD1-G93A mice

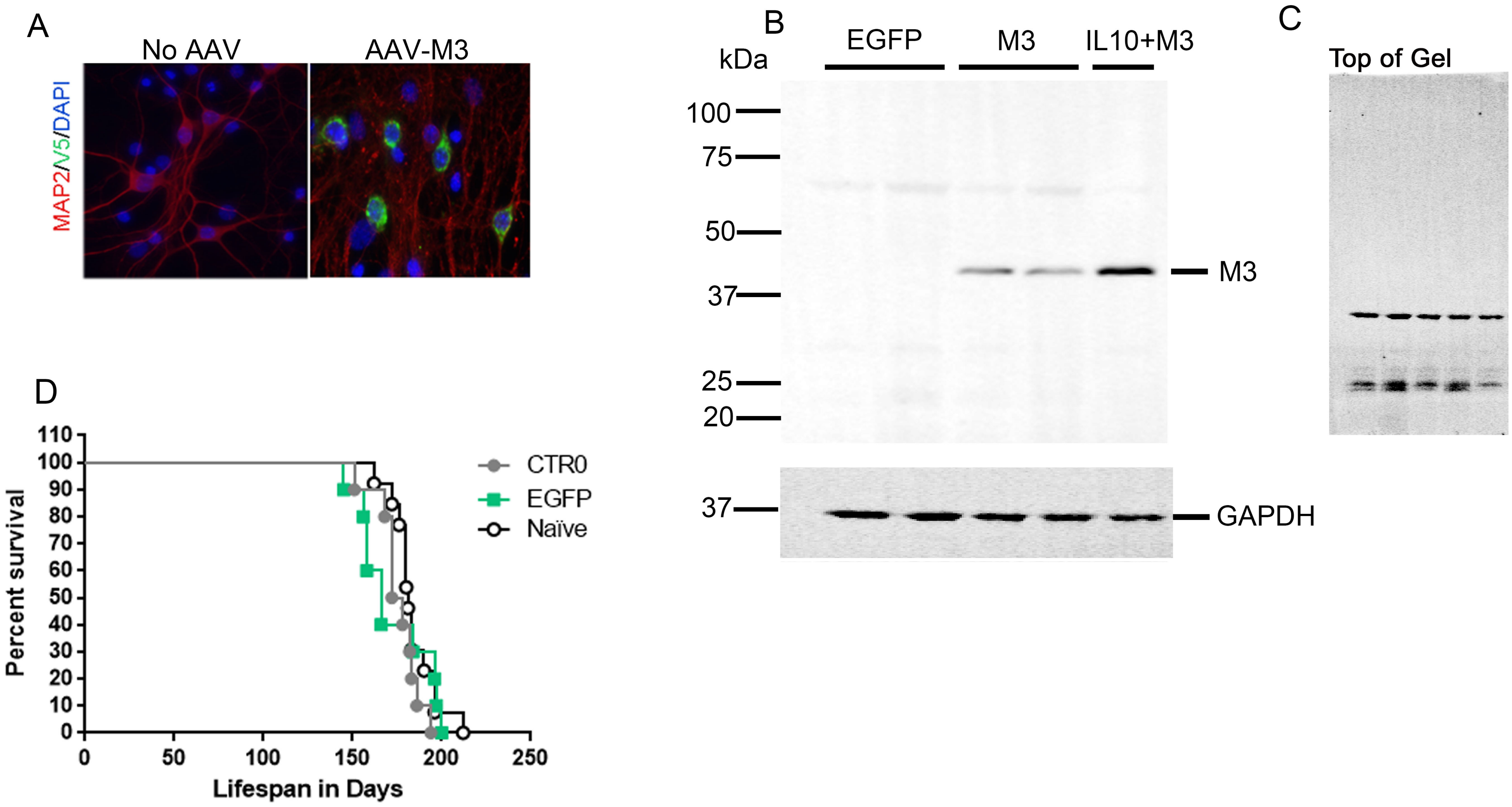

Median Age	AAV-CTR0	AAV-EGFP	Naïve
	181 d	177.5 d	181 d

Supplementary Figure S1. Validation of AAV-M3 expression and aggregated survival curves of control SOD1-G93A mice.

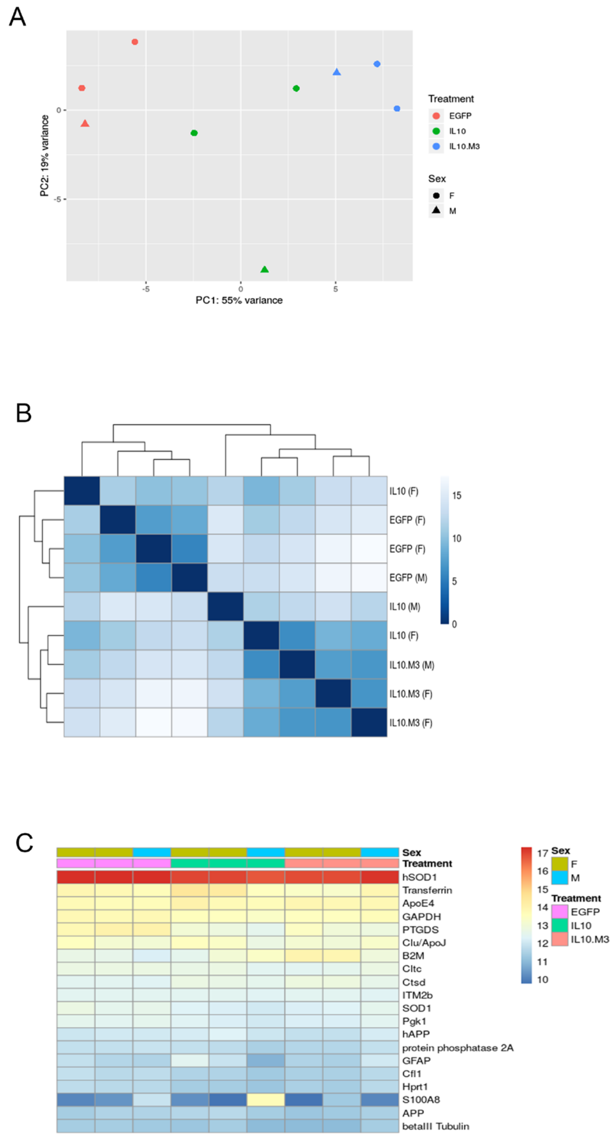

Supplementary Figure S2. Principle component analysis (PCA), sample-to-sample distance matrix and heat map of RNA analysis data from SOD1-G93A mice.

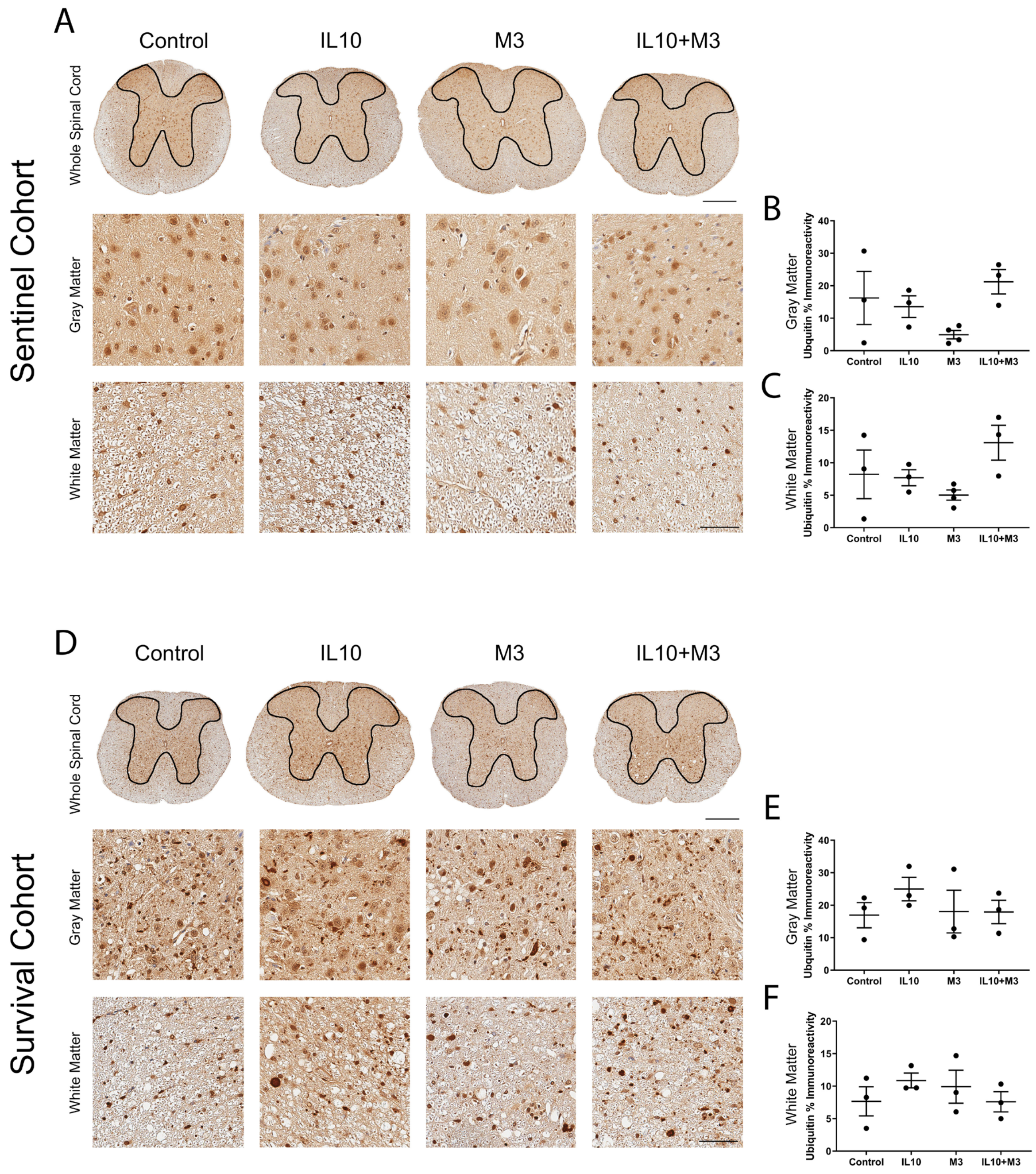

Supplementary Figure S3. Assessment of ubiquitin immunoreactivity burden in SOD1-G93A mice

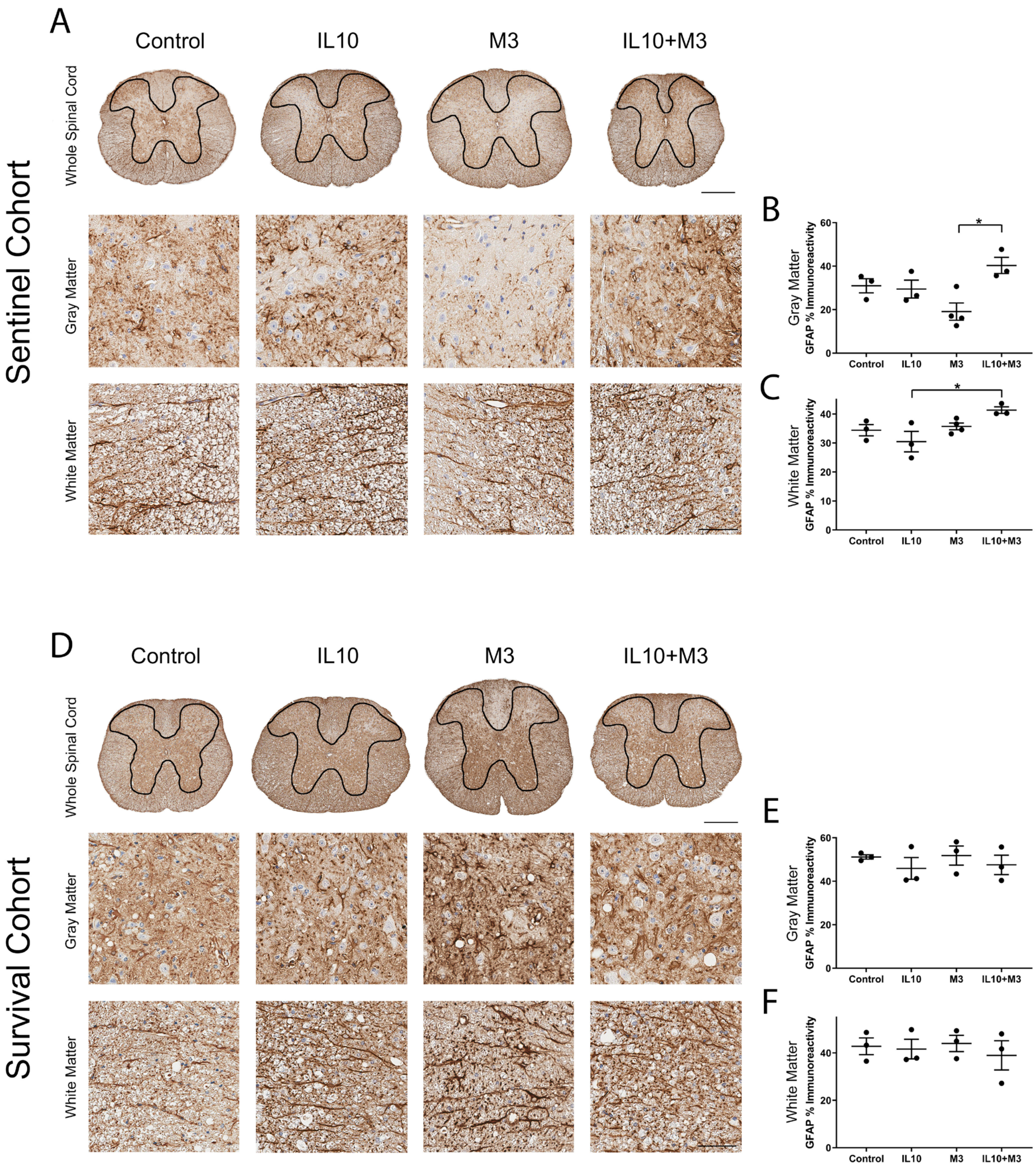

Supplementary Figure S4. Assessment of astrogliosis burden in SOD1-G93A mice

Table S1: Differential Expression of Genes in AAV-IL10 vs AAV-EGFP expressing SOD1-G93A mice

NanoString custom code-set readout. N=3 mice/group, basemean = mean normalized counts for all samples; lfcSE=standard error of fold change(log2), stat=Wald statistic; pvalue=Wald test p value; padj=Benjamini-Hochberg adjusted p value

	Row.names	baseMean	FoldChange (log2)	lfcSE	stat	pvalue	padj	accession$Accession
1	ABCA1	298.9425785	0.081644607	0.134330376	0.60778961	0.543327	0.794682229	NM_013454.3
2	ACE	124.4289786	-0.121923085	0.200091082	-0.609337928	0.5423	0.794682229	NM_009598.2
3	ADAM 10	847.2341408	-0.163566873	0.116142977	-1.408323409	0.159035	0.349146523	NM_007399.3
4	ADAM 17/TACE	278.6022788	-0.011110312	0.146172218	-0.076008373	0.939412	0.975150953	NM_001277266.1
5	Aif	261.4082923	-0.112006044	0.165619272	-0.676286296	0.498859	0.747295088	NM_012019.2
6	ALOX5	45.90395839	0.053619096	0.320032988	0.167542402	0.866943	0.950309662	NM_009662.2
7	ALOX5AP	63.62251462	0.41665663	0.345608974	1.205572369	0.227982	0.448913835	NM_009663.1
8	alpha-syn	235.3203746	-0.423094206	0.162146469	-2.609333449	0.009072	0.041255452	NM_000345.3
9	ApoE4	14220.98133	0.139295035	0.11874392	1.173070886	0.240767	0.464510805	NM_009696.2
10	APP	2899.648533	0.075782396	0.125218052	0.605203437	0.545044	0.794682229	NM_001198823.1
11	Aquaporin4	2176.041856	0.035549972	0.220730868	0.161055733	0.87205	0.950309662	NM_009700.2
12	Arc/arg3_1	48.23670775	0.071244356	0.287468261	0.247833816	0.804263	0.925386934	NM_018790.2
13	ARG1	18.20750062	-0.016796811	0.475723786	-0.035307907	0.971834	0.987692079	NM_007482.3
14	Axi	48.75439169	-0.085126965	0.267609128	-0.318101874	0.750408	0.899437162	NM_001159598.1
15	B2M	9222.488255	0.487694116	0.290606406	1.678194653	0.093309	0.229446471	NM_009735.3
16	BACE1	554.1522959	0.012563469	0.185005138	0.067908759	0.945858	0.976534751	NM_011792.4
17	BDNF	40.91004634	-0.480794594	0.350810606	-1.370524682	0.170523	0.365954232	NM_007540.4
18	betaIII Tubulin	2474.199911	-0.132491401	0.145196867	-0.912494901	0.361508	0.632512071	NM_023279.2
19	Bsn	110.5659627	0.031806477	0.209901333	0.151530612	0.879557	0.950309662	NM_007567.2
20	C1qa	1074.476466	1.308338565	0.190749811	6.858924555	6.94E-12	3.31E-10	NM_007572.2
21	C1qb	1553.339675	0.829389624	0.237347904	3.49440467	0.000475	0.004537398	NM_009777.2
22	C1r	151.5216614	0.276970932	0.321116816	0.86252391	0.388399	0.635091408	NM_023143.3
23	C1s	55.15804772	-0.313324747	0.375490868	-0.834440392	0.404033	0.637770885	NM_144938.2
24	c2	128.5216937	0.093289765	0.28845047	0.323416928	0.74638	0.899437162	NM_013484.2
25	C3	238.5235094	0.587942237	0.303800616	1.935289811	0.052955	0.148740525	NM_009778.2
26	C3ar1	110.949487	1.080388781	0.259318378	4.166263831	3.10E-05	0.000394265	NM_009779.2
27	c4a	1069.665379	1.407123236	0.233032697	6.038308147	1.56E-09	3.97E-08	NM_011413.2
28	C5AR1	73.0658107	0.446588826	0.285880442	1.562152426	0.118252	0.271333581	NM_007577.4
29	Calbindin	209.7170358	-0.269001848	0.172703645	-1.557592188	0.11933	0.271333581	NM_009788.4
30	CARD9	44.61882706	0.736710635	0.278977975	2.640748374	0.008272	0.038536874	NM_001037747.1
31	CASP1	83.33353754	-0.093696612	0.304216449	-0.307993246	0.758087	0.899437162	NM_009807.2
32	CASP8	68.15248793	0.034431999	0.27256748	0.126324676	0.899475	0.950309662	NM_009812.2
33	Catalase	890.8370753	-0.105100004	0.130929911	-0.802719587	0.422137	0.660886348	NM_009804.2
34	Ccl11	76.66942305	-1.257104195	0.470350881	-2.672694462	0.007524	0.036850633	NM_011330.3
35	Ccl17	23.39154374	-0.22425405	0.388576661	-0.577116621	0.563861	0.807809462	NM_011332.3
36	Ccl19	53.26199701	0.478860667	0.538547454	0.889170793	0.373911	0.635091408	NM_011888.2

37	CCL2	138.2659576	1.068104081	0.327750354	3.258895282	0.001118	0.008216449	NM_011333.3
38	Ccl21b	9.107440464	-0.052230498	0.86932319	-0.060081795	0.95209	NA	NM_011335.2
39	Ccl3	13.637989	-0.148700661	0.504529834	-0.294731156	0.768199	NA	NM_011337.2
40	Ccl4	9.089102494	0.105525111	0.602679177	0.175093342	0.861006	NA	NM_013652.2
41	CCL5	193.505276	1.533450878	0.500110158	3.066226215	0.002168	0.013367001	NM_013653.3
42	Ccl7	33.1338449	0.739587446	0.361140214	2.047923266	0.040568	0.127022887	NM_013654.3
43	Ccl8	257.818888	3.301616963	0.244229331	13.51851124	1.22E-41	2.32E-39	NM_021443.2
44	CCND1	89.89735751	-0.238257692	0.22897876	-1.040523112	0.298097	0.55278169	NM_007631.2
45	Ccr1	20.47612291	1.099425647	0.472498299	2.326835144	0.019974	0.076300837	NM_009912.4
46	Ccr4	5.487981912	0.03537904	0.818065351	0.043247205	0.965504	NA	NM_009916.2
47	CD14	32.16972651	-0.66096581	0.385627319	-1.714001518	0.086528	0.21745965	NM_009841.3
48	cd163	26.48868868	-0.665651299	0.525039338	-1.267812239	0.204865	0.420744268	NM_053094.2
49	CD33	21.74844118	0.368084155	0.390933719	0.941551309	0.346422	0.631308681	NM_001111058.1
50	CD36	55.26674877	-1.299249054	0.488061576	-2.6620597	0.007766	0.037084609	NM_001159555.1
51	CD39/ENTPD1	132.6882683	-0.091370946	0.206130452	-0.443267579	0.657572	0.866048543	NM_009848.3
52	Cd55	257.8240245	-0.77872916	0.197287362	-3.947182178	7.91E-05	0.000943974	NM_010016.2
53	Cd68	103.3113707	0.542800555	0.246768843	2.199631641	0.027833	0.098446495	NM_009853.1
54	Cebpd	12.00662518	-0.711166365	0.548964132	-1.295469639	0.195158	NA	NM_007679.4
55	Cfb	23.80206441	0.964083526	0.441115272	2.185559163	0.028848	0.100180795	NM_008198.2
56	Cfl1	3175.093576	-0.293912371	0.113341102	-2.593166692	0.00951	0.042240612	NM_007687.5
57	Chi3l3 (YM1)	84.04503679	2.855564135	1.172506844	2.435434941	0.014874	0.06002477	NM_009892.2
58	Cltc	6518.049991	-0.099608452	0.117744391	-0.845971945	0.397568	0.635091408	NM_001003908.1
59	Clu/ApoJ	9591.838654	-0.000790184	0.204616748	-0.003861777	0.996919	0.996918756	NM_013492.2
60	Connexin47	419.360947	-0.179363598	0.387859169	-0.462445166	0.643762	0.853878918	NM_080454.3
61	CR3/ITGAM	119.9596671	1.616695031	0.275942802	5.85880486	4.66E-09	9.89E-08	NM_001082960.1
62	CR4/ITGAX	4.648425355	0.537998829	0.897889198	0.599181759	0.549052	NA	NM_021334.2
63	Creb1	192.3398892	-0.418523235	0.152310275	-2.747833227	0.005999	0.031828301	NM_001037726.1
64	CRH	17.5378998	-0.554585166	0.467189756	-1.187066196	0.235202	NA	NM_205769.1
65	CRHR1	20.01612365	0.010959436	0.412999029	0.026536226	0.97883	0.987692079	NM_007762.4
66	Crp	11.72678113	-0.290056612	0.556522875	-0.521194412	0.602231	NA	NM_007768.4
67	Csf1	242.0925127	0.27649222	0.15467476	1.787571676	0.073845	0.195894743	NM_001113530.1
68	Csf1R/cd115	231.5484565	0.368142182	0.183063942	2.011003248	0.044325	0.132282761	NM_001037859.2
69	Ctsb	290.2324331	0.050428657	0.130753237	0.385678077	0.699735	0.885095393	NM_007798.2
70	Ctsc	447.1204377	1.040463254	0.180689654	5.758289052	8.50E-09	1.62E-07	NM_009982.4
71	Ctsd	6353.2232	0.214163653	0.106476828	2.011363946	0.044287	0.132282761	NM_009983.2
72	CX3CR1	280.7878462	0.614572914	0.250140868	2.456907255	0.014014	0.059481161	NM_009987.3
73	Cxcl10	102.2733474	1.233015263	0.41985532	2.936762274	0.003317	0.018351423	NM_021274.1
74	Cxcl5	16.38827824	-0.136898042	0.612043788	-0.223673608	0.823011	NA	NM_009141.2
75	Cxcl8	7.181298405	0.915930194	0.803921791	1.139327487	0.254567	NA	NM_011339.2
76	Cxcl9	173.1767982	0.600477046	0.552056301	1.087709794	0.276723	0.52330831	NM_008599.4

77	Cxcr4	52.34754286	0.256469131	0.465549838	0.550895115	0.581706	0.816954155	NM_009911.3
78	CXCR7	61.15461774	-0.494031255	0.271807708	-1.817576327	0.069129	0.185966511	NM_007722.3
79	Dock2	18.01498932	1.160865153	0.556715549	2.085203395	0.037051	NA	NM_033374.3
80	Drp1	918.6054013	-0.205596604	0.123105248	-1.670088052	0.094902	0.229446471	NM_001025947.1
81	ECE1	174.1161309	0.024421026	0.176586152	0.138295249	0.890007	0.950309662	NM_199307.2
82	ECE2	441.4016006	0.023112773	0.19066668	0.121220831	0.903516	0.950309662	NM_177942.1
83	eif2alpha	400.4314765	-0.379452716	0.139365066	-2.722724761	0.006475	0.033422922	NM_026114.3
84	Fcer1g	109.2290692	1.10950331	0.250898744	4.422115846	9.77E-06	0.000143601	NM_010185.4
85	FcgRI	122.8317285	0.811829447	0.250327122	3.243074266	0.001182	0.008364908	NM_010186.5
86	FcgRIIb	482.0005001	1.557144923	0.16426141	9.479675864	2.55E-21	2.44E-19	NM_001077189.1
87	FcgRIII	116.9542107	0.695566448	0.226893058	3.065613613	0.002172	0.013367001	NM_010188.5
88	FcgRIV	232.3686288	2.215451002	0.367552143	6.027582875	1.66E-09	3.97E-08	NM_144559.1
89	Fcrls	67.83999876	1.83899792	0.400470688	4.592091192	4.39E-06	7.62E-05	NM_030707.3
90	FcRn	291.3638239	-0.338646952	0.163181089	-2.075283074	0.03796	0.120840311	NM_010189.3
91	Flii	302.5001484	0.111239152	0.131895255	0.843390097	0.39901	0.635091408	NM_022009.1
92	Fos	69.6340725	0.087203517	0.281015434	0.310315755	0.756321	0.899437162	NM_010234.2
93	FTL	1378.703858	-0.15950472	0.09021759	-1.768000222	0.077061	0.200035469	NM_010240.2
94	GAPDH	12836.04364	-0.311633057	0.090727118	-3.434839165	0.000593	0.005392618	NM_001001303.1
95	GBA	194.6803465	0.096796856	0.17242031	0.561400543	0.574525	0.812845794	NM_001077411.1
96	GFAP	3510.793856	0.216135379	0.321045485	0.673223544	0.500805	0.747295088	NM_001131020.1
97	Gls	1000.139973	-0.101206018	0.113502645	-0.891662194	0.372574	0.635091408	NM_001081081.2
98	GRIN2a	121.8486106	0.120443743	0.210247676	0.57286599	0.566735	0.807809462	NM_008170.2
99	GRN	646.3594499	0.487353246	0.159368319	3.058030915	0.002228	0.013367001	NM_008175.3
100	Gusb	142.2564398	0.078810931	0.169238805	0.465678846	0.641445	0.853878918	NM_010368.1
101	H2-Eb1	144.443374	-0.21508462	0.538276657	-0.399580063	0.689466	0.885095393	NM_010382.2
102	H2Ea	224.1681037	0.042717987	1.949833099	0.021908535	0.982521	0.987692079	NM_010381.2
103	hApoE4	8.412064619	-0.064627188	0.70298093	-0.09193306	0.926751	NA	NM_000041.2
104	hAPP	4308.511156	0.08727067	0.125747004	0.694017887	0.487671	0.745161275	NM_000484.3
105	Hck	22.85234665	1.209656084	0.561659074	2.153719473	0.031262	0.10475572	NM_001172117.1
106	hFTL	9.645873524	0.562895416	0.586679491	0.959459849	0.337327	NA	NM_000146.3
107	hGRN	3.003641394	-0.658158703	1.181278442	-0.557157974	0.57742	NA	NM_002087.2
108	hITM2b	4.2048416	-0.152168719	0.998565613	-0.152387302	0.878881	NA	NM_021999.2
109	hLrrk2	19.68048002	-0.083657657	0.429430352	-0.194810768	0.845541	0.950309662	NM_198578.2
110	HO-1	84.22307512	-0.069315139	0.304205862	-0.227856027	0.819758	0.931986966	NM_010442.2
111	Hprt1	3011.15463	-0.385363857	0.107655252	-3.579610386	0.000344	0.00345918	NM_013556.2
112	hSOD1	142407.46	-0.401990537	0.121963902	-3.295979633	0.000981	0.007493245	NM_000454.4
113	Hspb1	317.5033807	-0.037358348	0.207584059	-0.179967326	0.857178	0.950309662	NM_013560.2
114	Hspb2	71.68351457	-0.649279614	0.307932292	-2.108514212	0.034987	0.113261494	NM_024441.2
115	hTDP43	4.7269634	-0.08613785	0.830896872	-0.103668521	0.917432	NA	NM_007375.3
116	IDE	331.7957386	-0.056188354	0.217339789	-0.258527693	0.796	0.925386934	NM_031156.2

117	IFIH1	62.59499796	0.156976859	0.322149384	0.487279713	0.62606	0.848067258	NM_001164477.1
118	IFN-gamma	34.18428356	0.131061203	0.319014763	0.410831153	0.681196	0.885091853	NM_008337.3
119	IGF1	66.22956731	0.149124294	0.41640553	0.358122751	0.720251	0.899437162	NM_001111274.1
120	IGFBP2	285.4946982	-0.440538254	0.249543633	-1.765375652	0.077501	0.200035469	NM_008342.3
121	IGFBP3	234.212813	-0.327705858	0.243370035	-1.346533304	0.178131	0.378032691	NM_008343.2
122	IGFBP4	382.4101308	-0.047522992	0.196954806	-0.241288816	0.809331	0.925642361	NM_010517.3
123	IGFBP5	199.3597806	-0.595026826	0.302820371	-1.964949795	0.04942	0.140884019	NM_010518.2
124	IL-10	2004.206112	7.3875376	0.986948469	7.485231331	7.14E-14	4.55E-12	NM_010548.1
125	IL-1b	29.36938877	1.595050752	0.521858114	3.05648357	0.002239	0.013367001	NM_008361.3
126	IL-4	6.418980022	-0.492450446	0.734529936	-0.670429375	0.502584	NA	NM_021283.2
127	IL-6	17.23951367	-0.367702023	0.992756912	-0.370384752	0.711096	NA	NM_031168.1
128	Il10rb	190.3887088	-0.049539518	0.164195997	-0.301709659	0.762873	0.899437162	NM_008349.5
129	Il12a	5.456862028	0.069411204	0.728424382	0.095289512	0.924085	NA	NM_008351.2
130	Il1a	9.014357529	0.328391915	0.630373173	0.52094843	0.602403	NA	NM_010554.4
131	Il1r1	72.10115563	-0.376267241	0.22495527	-1.672631371	0.0944	0.229446471	NM_001123382.1
132	Il1rn	16.14253541	0.588544366	0.718287442	0.819371648	0.412574	NA	NM_031167.5
133	Il6ra	40.98353125	-0.092181053	0.29739502	-0.309961655	0.75659	0.899437162	NM_010559.2
134	Il7	17.84636372	-0.349640652	0.452444076	-0.772782031	0.439651	NA	NM_008371.2
135	Il8rb	13.91011996	0.435758317	0.826413122	0.52728872	0.597993	NA	NM_009909.3
136	Itgb2/MAC-1	93.23649837	1.051868782	0.35869779	2.932465187	0.003363	0.018351423	NM_008404.4
137	ITM2b	5586.73382	-0.022727236	0.068774224	-0.33046155	0.741051	0.899437162	NM_008410.2
138	JAK3	5.375063014	-0.277802162	0.846417932	-0.328209212	0.742753	NA	NM_001190830.1
139	Limk1	482.0943809	-0.114531788	0.131909158	-0.868262594	0.385251	0.635091408	NM_010717.2
140	Lrp1	900.7427626	0.043100284	0.111237685	0.387461173	0.698415	0.885095393	NM_008512.2
141	Lrrk2	79.02689395	-0.024214597	0.212465485	-0.113969557	0.909262	0.950309662	NM_025730.3
142	Ltbr	62.23137133	-0.15148706	0.288920465	-0.524320974	0.600055	0.835129077	NM_010736.3
143	Ly86	639.6183313	0.888220123	0.2125711	4.178461343	2.93E-05	0.000394265	NM_010745.2
144	Ly96	43.08047647	0.199165248	0.337115146	0.590792938	0.554659	0.802575007	NM_001159711.1
145	Lysozyme M (Lyz2)	454.0167746	-0.282577338	0.412460558	-0.685101479	0.49328	0.747295088	NM_017372.3
146	Maff	17.33136134	-0.797488194	0.475951743	-1.675565235	0.093823	NA	NM_010755.3
147	Mafk	35.9816885	0.041647388	0.30127816	0.138235668	0.890054	0.950309662	NM_010757.2
148	Map2k4	1425.046386	-0.155506303	0.111229123	-1.39807183	0.162091	0.351812229	NM_009157.4
149	Map2k6	56.0004215	-0.385256961	0.30319471	-1.270658584	0.20385	0.420744268	NM_011943.2
150	Mapk8	490.470852	-0.380778959	0.114784118	-3.317348821	0.000909	0.007232221	NM_016700.3
151	Mapt	382.1418255	-0.005319876	0.154473581	-0.034438741	0.972527	0.987692079	NM_001038609m.1
152	Maptex1	10.48602398	0.436564529	0.573938444	0.760646954	0.446868	NA	NM_005910ex1.1
153	Maptex10	40.35247636	-0.608102498	0.662551357	-0.917819414	0.358713	0.632512071	NM_005910ex10.1
154	Maptex9_11	72.62810261	-0.240891494	0.282669208	-0.852202813	0.394102	0.635091408	NM_001038609ex9_11.1
155	MARCO	26.34607934	1.847642721	0.853613755	2.164495018	0.030426	0.103775684	NM_010766.2
156	Masp2	7.144244426	0.724545527	0.786166688	0.921618198	0.356728	NA	NM_010767.3

157	Mbl2	3.49472495	0.038907455	0.99278159	0.039190347	0.968739	NA	NM_010776.1
158	Mef2b	4.84848241	0.430481298	0.930031839	0.46286727	0.64346	NA	NM_008578.2
159	Mef2c	155.5094029	-0.02294628	0.204148084	-0.112400175	0.910506	0.950309662	NM_001170537.1
160	Mertk	70.22898927	-0.240067622	0.257061539	-0.93389164	0.35036	0.631308681	NM_008587.1
161	Mfn1	261.2357485	-0.124734499	0.16191303	-0.770379622	0.441075	0.684920934	NM_024200.4
162	MMP11	25.80271997	0.097752991	0.389639998	0.250880277	0.801907	0.925386934	NM_008606.2
163	MMP2	95.51245963	-0.970919521	0.314459417	-3.087582906	0.002018	0.013367001	NM_008610.2
164	MMP3	4.634224081	-0.872787825	0.843018817	-1.035312388	0.300523	NA	NM_010809.1
165	MMP9	52.11905005	0.378864342	0.511638761	0.740491868	0.459002	0.707010519	NM_013599.2
166	Mrc1	166.0972246	0.293710042	0.255946739	1.147543596	0.251157	0.47970992	NM_008625.2
167	Ms4a4a	15.89770443	-0.48925374	0.557837714	-0.877053895	0.380457	NA	XM_003086124.1
168	Ms4a6a	91.90397713	1.372524776	0.223583381	6.138760259	8.32E-10	2.65E-08	NM_026835.2
169	Msr1	33.9077417	0.3347033	0.356576343	0.938658178	0.347906	0.631308681	NM_001113326.1
170	Myc	44.91013758	0.299081801	0.324769683	0.920904313	0.3571	0.632512071	NM_010849.4
171	Myeloperoxidase	112.7696334	1.483414633	0.67258674	2.205536543	0.027416	0.098446495	NM_010824.2
172	Myl2	16.35784745	0.074161799	0.500016845	0.1483186	0.882091	NA	NM_010861.3
173	Ncf1	106.0048408	0.599544707	0.327684671	1.829639163	0.067304	0.183643536	NM_010876.3
174	Neprilysin	152.8413994	-0.516952155	0.207784262	-2.487927384	0.012849	0.055776326	NM_008604.3
175	NLRP3	11.31601646	-0.163589785	0.766213745	-0.213504111	0.830934	NA	NM_145827.3
176	Nos2	3.116247036	0.544936871	1.186553127	0.459260406	0.646047	NA	NM_010927.3
177	NPY	209.2460409	-0.435268244	0.21824791	-1.994375316	0.046111	0.133442596	NM_023456.2
178	P2ry12	266.3176122	-0.514849531	0.211842792	-2.430337732	0.015085	0.06002477	NM_027571.3
179	P2RY6	37.65234101	0.69622351	0.327432682	2.126310381	0.033477	0.110244621	NM_183168.2
180	Park9 (ATP13A2)	45.27334907	-0.081737193	0.2810307	-0.290847916	0.771168	0.903638151	NM_001164366.1
181	Peripherin	445.6879727	-0.468264944	0.232297084	-2.015802077	0.043821	0.132282761	NM_001163588.1
182	Pgk1	5301.183181	-0.260645187	0.116584769	-2.235671	0.025373	0.093198193	NM_008828.2
183	Pink1	1288.600065	-0.425322609	0.128068278	-3.321061346	0.000897	0.007232221	NM_026880.2
184	Plasmin	5.509152618	-0.422353933	0.774334163	-0.545441429	0.58545	NA	NM_008877.3
185	PPP1R2	759.2491201	-0.265879269	0.141617123	-1.877451423	0.060456	0.167349907	NM_025800.3
186	Prkcb1	660.8364374	0.019015087	0.148679904	0.127892782	0.898234	0.950309662	NM_008855.2
187	protein phosphatas	3518.176001	-0.168647145	0.13616785	-1.238523961	0.215522	0.433312337	NM_019411.4
188	PSD95	450.3996488	0.056588948	0.129451002	0.437145692	0.662006	0.866048543	NM_001109752.1
189	PTAFR/PAFR	71.44067685	-0.088375338	0.258327913	-0.34210526	0.732272	0.899437162	NM_001081211.1
190	PTGDS	10847.82796	-1.168435253	0.185191928	-6.309320631	2.80E-10	1.07E-08	NM_008963.2
191	PTGER1	10.50446251	0.222242425	0.603323731	0.368363473	0.712602	NA	NM_013641.2
192	PTGER4	15.01548642	0.11730294	0.558732956	0.209944552	0.833711	NA	NM_008965.1
193	PTGS2	24.15905155	0.201668882	0.413266409	0.487987598	0.625559	0.848067258	NM_011198.3
194	Ptpn6	121.0241539	1.096644765	0.293843798	3.732067081	0.00019	0.00213375	NM_013545.2
195	PTPRC/CD45	149.5957074	0.907187565	0.30501165	2.974271848	0.002937	0.016998118	NM_011210.3
196	S100A11	628.0088937	0.202598518	0.230819523	0.877735623	0.380087	0.635091408	NM_016740.3

197	S100A8	2924.983337	1.282576129	1.082420444	1.184914915	NA	NA	NM_013650.2
198	S100A9	366.1141923	1.177480454	1.063170307	1.107518189	NA	NA	NM_009114.2
199	SERPINA1	18.10955516	1.253419934	0.806870107	1.55343459	0.120319	NA	NM_009243.3
200	SERPINA3	197.6117204	-0.466407252	0.233134554	-2.000592555	0.045436	0.133442596	NM_009252.2
201	SerpinG1	149.7404413	-0.374878009	0.309320458	-1.211940561	0.225535	0.448720944	NM_009776.3
202	Snap91	1264.625299	0.017765871	0.123737715	0.143576848	0.885835	0.950309662	NM_013669.1
203	SOD1	5543.260045	-0.433763534	0.117481812	-3.692176062	0.000222	0.002359311	NM_011434.1
204	Sox10	218.8138429	0.031967193	0.198604043	0.160959426	0.872125	0.950309662	XM_128139.6
205	STAT3	553.9633604	0.264756508	0.108109194	2.4489731	0.014326	0.059485765	NM_213659.2
206	STAT6	112.4091062	-0.22478565	0.24776561	-0.907251212	0.364274	0.632512071	NM_009284.2
207	synaptophysin	1026.354232	-0.117193967	0.112278045	-1.043783468	0.296586	0.55278169	NM_009305.2
208	Synuclein	139.4461099	-0.143780963	0.168379668	-0.853909291	0.393155	0.635091408	NM_009221.2
209	TDP43	737.348434	-0.21411757	0.14573589	-1.469216474	0.141774	0.314870348	NM_001003899.2
210	Tgfb1	98.33748139	0.42069223	0.262665919	1.601624726	0.109239	0.254446053	NM_011577.1
211	Tgfb3	93.56036915	-0.335411896	0.284327231	-1.179668566	0.238132	0.464114526	NM_009368.2
212	Tgfbr1	228.6382337	-0.325595612	0.14552347	-2.23740962	0.02526	0.093198193	NM_009370.2
213	TIMP1	70.0877462	-0.858888687	0.359401398	-2.389775585	0.016859	0.06571441	NM_001044384.1
214	Tlr1	22.05724811	0.674562656	0.417639691	1.615178516	0.106272	0.250592098	NM_030682.1
215	Tlr2	22.35792179	-0.053591568	0.427338178	-0.125407864	0.900201	0.950309662	NM_011905.2
216	Tlr4	49.12525534	-0.119523596	0.303135469	-0.394291031	0.693366	0.885095393	NM_021297.2
217	Tlr6	25.78665244	0.781634601	0.445326079	1.75519611	0.079226	0.201761625	NM_011604.3
218	Tlr7	21.19085089	0.239976549	0.467546586	0.513267674	0.607764	0.835129077	NM_133211.3
219	TLr8	6.669783698	0.040805158	0.804569068	0.050716787	0.959551	NA	NM_133212.2
220	TLR9	24.06271357	0.681000179	0.409393153	1.663438124	0.096225	0.229736632	NM_031178.2
221	TNFa	3.672929281	-0.014459364	1.106199327	-0.013071211	0.989571	NA	NM_013693.2
222	TNFRSF1A	142.8534306	0.063790046	0.19329895	0.330007205	0.741395	0.899437162	NM_011609.4
223	TNFRSF1B	7.303172696	0.430465644	0.676748444	0.636079251	0.524725	NA	NM_011610.3
224	TNFSF11	6.579446478	-0.078077427	0.811512324	-0.096212251	0.923352	NA	NM_011613.3
225	TNFSF9	6.43442612	0.209169874	0.678739441	0.308174038	0.75795	NA	NM_009404.3
226	tPA	251.9357224	-0.051047302	0.166814138	-0.306013042	0.759595	0.899437162	NM_008872.2
227	Transferrin	15023.96921	0.380270251	0.258604323	1.470471362	0.141434	0.314870348	NM_133977.2
228	Trem-1	5.71442201	0.169921994	0.875689817	0.194043587	0.846142	NA	NM_021406.5
229	Trem-2	127.4788744	0.976623176	0.21951395	4.449025569	8.63E-06	0.000137298	NM_031254.2
230	TREMl1	5.622078661	0.923126311	0.78280787	1.179250167	0.238299	NA	NM_027763.1
231	TREMl2	8.892712436	1.337170681	0.74084738	1.804920577	0.071087	NA	NM_001033405.2
232	TREMl4	9.110483546	0.642054239	0.678350839	0.946492879	0.343897	NA	NM_001033922.2
233	Tubb5	1268.900549	-0.124358227	0.099234468	-1.253175731	0.210142	0.426990172	NM_011655.5
234	TyroBP/DAP12	799.9628722	0.681591302	0.204412396	3.334393189	0.000855	0.007232221	NM_011662.2
235	Tyrosine hydroxylas	23.24362861	-0.499636606	0.381883349	-1.308348759	0.190755	0.400375971	NM_009377.1
236	UbC	2443.363832	-0.050672069	0.108682183	-0.466240809	0.641043	0.853878918	NM_019639.4

237	Ubiquilin1/2	174.8371541	-0.171478909	0.193598655	-0.885744322	0.375755	0.635091408	NM_152234.2
238	VWF	219.6479321	-0.009484892	0.189981212	-0.04992542	0.960182	0.985993159	NM_011708.3
239	ZBP1	113.7489311	1.254068127	0.467729323	2.681183465	0.007336	0.036850633	NM_021394.2
240	zif268	72.61017483	0.160959004	0.312876225	0.514449456	0.606938	0.835129077	NM_007913.5

Table S2: Differential Expression of Genes in AAV-IL10+AAV-M3 vs AAV-EGFP expressing SOD1-G93A mice

NanoString custom code-set readout. N=3 mice/group, basemean = mean normalized counts for all samples; lfcSE=standard error of fold change(log2), stat=Wald statistic; pvalue=Wald test p value; padj=Benjamini-Hochberg adjusted p value

	Row.names	baseMean	log2FoldChange	lfcSE	stat	pvalue	padj	accession$Accession
1	ABCA1	298.9425785	0.071938276	0.133616555	0.538393437	0.590305456	0.747485331	NM_013454.3
2	ACE	124.4289786	-0.438899359	0.201798265	-2.17494119	0.029634524	0.133075786	NM_009598.2
3	ADAM 10	847.2341408	-0.231181848	0.115969833	-1.993465384	0.04621051	0.177388733	NM_007399.3
4	ADAM 17/TACE	278.6022788	-0.148347612	0.146157908	-1.014981764	0.31011452	0.554941773	NM_001277266.1
5	Aif	261.4082923	-0.170567051	0.165184102	-1.032587578	0.301796944	0.554941773	NM_012019.2
6	ALOX5	45.90395839	-0.317919996	0.323678539	-0.982209068	0.325996861	0.562226471	NM_009662.2
7	ALOX5AP	63.62251462	0.336852683	0.345042868	0.976263283	0.32893401	0.563210751	NM_009663.1
8	alpha-syn	235.3203746	-0.342729244	0.160717214	-2.132498657	0.032965875	0.140104968	NM_000345.3
9	ApoE4	14220.98133	0.151632293	0.118724294	1.277179989	0.20153871	0.432128045	NM_009696.2
10	APP	2899.648533	-0.173265103	0.125285885	-1.38295789	0.166677778	0.404230901	NM_001198823.1
11	Aquaporin4	2176.041856	0.034295121	0.220666455	0.155416104	0.876493289	0.941895215	NM_009700.2
12	Arc/arg3_1	48.23670775	-0.17446105	0.289202501	-0.603248758	0.546343216	0.734630991	NM_018790.2
13	ARG1	18.20750062	0.716879327	0.453505232	1.580752054	0.113934741	0.327337363	NM_007482.3
14	Axi	48.75439169	-0.054790354	0.264634861	-0.207041333	0.835977579	0.926264686	NM_001159598.1
15	B2M	9222.488255	1.157876522	0.290546508	3.985167567	6.74E-05	0.000668706	NM_009735.3
16	BACE1	554.1522959	-0.295206936	0.185384307	-1.592405212	0.111293686	0.327011077	NM_011792.4
17	BDNF	40.91004634	-0.084794043	0.34162832	-0.248205545	0.803975376	0.918883767	NM_007540.4
18	betaIII Tubulin	2474.199911	-0.194556616	0.145146033	-1.340419795	0.180108904	0.410164508	NM_023279.2
19	Bsn	110.5659627	-0.014912485	0.209005407	-0.071349757	0.943119396	0.967731326	NM_007567.2
20	C1qa	1074.476466	1.544671387	0.1904224	8.111815552	4.99E-16	2.37E-14	NM_007572.2
21	C1qb	1553.339675	1.44306208	0.236961689	6.089853964	1.13E-09	2.69E-08	NM_009777.2
22	C1r	151.5216614	0.49531999	0.319586854	1.549875987	0.121171284	0.331480065	NM_023143.3
23	C1s	55.15804772	0.058335504	0.369692579	0.157794632	0.874618629	0.941895215	NM_144938.2
24	c2	128.5216937	0.447455434	0.285595278	1.566746611	0.117173931	0.327491486	NM_013484.2
25	C3	238.5235094	0.41111499	0.303902256	1.352786897	0.17612372	0.410164508	NM_009778.2
26	C3ar1	110.949487	1.336256237	0.256794686	5.203597701	1.95E-07	2.74E-06	NM_009779.2
27	c4a	1069.665379	1.468633088	0.23288928	6.306142941	2.86E-10	7.57E-09	NM_011413.2
28	C5AR1	73.0658107	0.235913544	0.286585026	0.82318866	0.410400717	0.614310507	NM_007577.4
29	Calbindin	209.7170358	-0.27139821	0.17179409	-1.579787815	0.114155467	0.327337363	NM_009788.4
30	CARD9	44.61882706	0.371361347	0.282609338	1.314044858	0.188831109	0.419261823	NM_001037747.1
31	CASP1	83.33353754	0.416483918	0.298525251	1.395137988	0.162974233	0.404230901	NM_009807.2
32	CASP8	68.15248793	0.265306679	0.268204846	0.989194201	0.322568135	0.56037384	NM_009812.2
33	Catalase	890.8370753	-0.240440775	0.130920031	-1.83654688	0.066276815	0.230043054	NM_009804.2
34	Ccl11	76.66942305	-0.673736048	0.464276183	-1.451153586	0.146737098	0.375520745	NM_011330.3
35	Ccl17	23.39154374	-0.488745239	0.391935928	-1.247002902	0.212396433	0.443424133	NM_011332.3
36	Ccl19	53.26199701	0.467059132	0.537637517	0.868724962	0.384997585	0.600763022	NM_011888.2
37	CCL2	138.2659576	1.803291603	0.324170086	5.562794603	2.65E-08	5.27E-07	NM_011333.3
38	Ccl21b	9.107440464	-0.209197119	0.869821246	-0.240505874	0.80993811	0.918883767	NM_011335.2
39	Ccl3	13.637989	-0.079779995	0.497528998	-0.160352453	0.872603442	0.941895215	NM_011337.2
40	Ccl4	9.089102494	0.344139488	0.587727249	0.58554285	0.558182763	0.736685574	NM_013652.2
41	CCL5	193.505276	2.653703865	0.497710805	5.331818874	9.72E-08	1.45E-06	NM_013653.3
42	Ccl7	33.1338449	0.870085015	0.356779994	2.438715816	0.014739554	0.076261173	NM_013654.3

43	Ccl8	257.818888	3.895836177	0.242354291	16.07496268	3.82E-58	9.10E-56	NM_021443.2
44	CCND1	89.89735751	0.146117911	0.223077605	0.655009324	0.512461751	0.713250858	NM_007631.2
45	Ccr1	20.47612291	0.910224024	0.473454106	1.922517962	0.054540616	0.202822915	NM_009912.4
46	Ccr4	5.487981912	0.730811127	0.77723376	0.94027198	0.347078068	0.568043242	NM_009916.2
47	CD14	32.16972651	-0.534971229	0.379977243	-1.40790334	0.159159694	0.4029788	NM_009841.3
48	cd163	26.48868868	-0.852521188	0.525971572	-1.620850314	0.105049745	0.316478978	NM_053094.2
49	CD33	21.74844118	0.344281623	0.388114733	0.887061461	0.375045811	0.595072687	NM_001111058.1
50	CD36	55.26674877	-1.472945376	0.488489969	-3.015303221	0.002567225	0.017457127	NM_001159555.1
51	CD39/ENTPD1	132.6882683	0.223485485	0.202406726	1.104140608	0.269532149	0.525808618	NM_009848.3
52	Cd55	257.8240245	-0.642502691	0.195806448	-3.281315285	0.001033242	0.008197051	NM_010016.2
53	Cd68	103.3113707	1.036333712	0.241855592	4.28492765	1.83E-05	0.000197755	NM_009853.1
54	Cebpd	12.00662518	-0.936844002	0.552641447	-1.695211255	0.090035353	0.280481672	NM_007679.4
55	Cfb	23.80206441	1.45632406	0.429831303	3.388129365	0.000703711	0.005775281	NM_008198.2
56	Cfl1	3175.093576	-0.229415262	0.113204994	-2.026547196	0.042708741	0.169961144	NM_007687.5
57	Chi3l3 (YM1)	84.04503679	-0.520357718	1.184358515	-0.439358279	0.660401952	0.798156843	NM_009892.2
58	Cltc	6518.049991	-0.16159555	0.117720755	-1.372702292	0.169844917	0.404230901	NM_001003908.1
59	Clu/ApoJ	9591.838654	-0.09324142	0.204611198	-0.455700477	0.648605379	0.798156843	NM_013492.2
60	Connexin47	419.360947	-0.386021709	0.387956893	-0.995011858	0.319730497	0.55952837	NM_080454.3
61	CR3/ITGAM	119.9596671	1.547504657	0.275569622	5.61565765	1.96E-08	4.24E-07	NM_001082960.1
62	CR4/ITGAX	4.648425355	0.745774486	0.881217679	0.846299959	0.397385431	0.606072879	NM_021334.2
63	Creb1	192.3398892	-0.310928577	0.150227253	-2.069721511	0.038478431	0.160664327	NM_001037726.1
64	CRH	17.5378998	-0.157126901	0.450697901	-0.348630203	0.72736695	0.856996703	NM_205769.1
65	CRHR1	20.01612365	-0.320688515	0.418396784	-0.766469839	0.443396791	0.651410101	NM_007762.4
66	Crp	11.72678113	-0.525196598	0.560165632	-0.93757376	0.348463502	0.568043242	NM_007768.4
67	Csf1	242.0925127	0.015889063	0.1553738	0.102263462	0.918547556	0.960247484	NM_001113530.1
68	Csf1R/cd115	231.5484565	0.337612889	0.182526952	1.849660482	0.0643625	0.228630969	NM_001037859.2
69	Ctsb	290.2324331	0.176164365	0.129216474	1.363327441	0.172779338	0.407143389	NM_007798.2
70	Ctsc	447.1204377	1.400065181	0.179552574	7.797522181	6.31E-15	2.15E-13	NM_009982.4
71	Ctsd	6353.2232	0.339123108	0.10639391	3.187429692	0.001435434	0.010654279	NM_009983.2
72	CX3CR1	280.7878462	0.648569568	0.249650247	2.59791278	0.009379231	0.054445295	NM_009987.3
73	Cxcl10	102.2733474	2.217761375	0.414977412	5.344294195	9.08E-08	1.44E-06	NM_021274.1
74	Cxcl5	16.38827824	-0.35761026	0.613958462	-0.582466538	0.560252474	0.736685574	NM_009141.2
75	Cxcl8	7.181298405	0.479998146	0.8139108	0.589742937	0.555363004	0.736685574	NM_011339.2
76	Cxcl9	173.1767982	2.140791813	0.54832986	3.904204327	9.45E-05	0.000899982	NM_008599.4
77	Cxcr4	52.34754286	-0.205675594	0.468505938	-0.439003175	0.660659236	0.798156843	NM_009911.3
78	CXCR7	61.15461774	-0.26818216	0.266318339	-1.00699847	0.313935509	0.55758695	NM_007722.3
79	Dock2	18.01498932	1.2965295	0.551714332	2.350001487	0.018773336	0.093084458	NM_033374.3
80	Drp1	918.6054013	-0.164188893	0.122728634	-1.337820585	0.18095493	0.410164508	NM_001025947.1
81	ECE1	174.1161309	-0.314425695	0.178088013	-1.765563498	0.077469108	0.249157403	NM_199307.2
82	ECE2	441.4016006	-0.232340727	0.190981194	-1.216563377	0.223770384	0.455191037	NM_177942.1
83	eif2alpha	400.4314765	-0.17076068	0.137854875	-1.238698887	0.215457031	0.444143825	NM_026114.3
84	Fcer1g	109.2290692	1.328767103	0.248502355	5.347100653	8.94E-08	1.44E-06	NM_010185.4
85	FcgRI	122.8317285	1.131891773	0.247485517	4.573567725	4.79E-06	5.43E-05	NM_010186.5
86	FcgRIIb	482.0005001	1.853021209	0.163303401	11.3471073	7.67E-30	9.12E-28	NM_001077189.1
87	FcgRIII	116.9542107	1.084730812	0.223037195	4.86345254	1.15E-06	1.53E-05	NM_010188.5
88	FcgRIV	232.3686288	3.023124705	0.365658133	8.26762605	1.37E-16	8.13E-15	NM_144559.1

89	Fcrls	67.83999876	-0.632136362	0.42426428	-1.489958952	0.136235029	0.364313898	NM_030707.3
90	FcRn	291.3638239	-0.170916702	0.161628823	-1.057464246	0.290299764	0.546203978	NM_010189.3
91	Flii	302.5001484	-0.124730863	0.132436112	-0.941819126	0.346285252	0.568043242	NM_022009.1
92	Fos	69.6340725	-0.106735754	0.281635712	-0.378985156	0.704698887	0.834419577	NM_010234.2
93	FTL	1378.703858	-0.117718103	0.089878512	-1.309746909	0.190281469	0.419261823	NM_010240.2
94	GAPDH	12836.04364	-0.238359165	0.090683098	-2.628485036	0.008576613	0.051418648	NM_001001303.1
95	GBA	194.6803465	-0.228254578	0.17367643	-1.314251901	0.188761448	0.419261823	NM_001077411.1
96	GFAP	3510.793856	0.065500808	0.321047421	0.204022221	0.838336134	0.926264686	NM_001131020.1
97	Gls	1000.139973	-0.208008513	0.113434259	-1.833736253	0.066693154	0.230043054	NM_001081081.2
98	GRIN2a	121.8486106	-0.029832372	0.210338989	-0.141829966	0.887214314	0.946892407	NM_008170.2
99	GRN	646.3594499	0.517362376	0.159034068	3.253154382	0.001141315	0.008762352	NM_008175.3
100	Gusb	142.2564398	0.075224575	0.167993533	0.447782568	0.654310125	0.798156843	NM_010368.1
101	H2-Eb1	144.443374	0.423727124	0.536018089	0.790509002	0.429230567	0.638480469	NM_010382.2
102	H2Ea	224.1681037	2.510677168	1.948210818	1.288709181	0.197499209	0.427316471	NM_010381.2
103	hApoE4	8.412064619	0.520422971	0.675702619	0.770195284	0.441184061	0.651410101	NM_000041.2
104	hAPP	4308.511156	-0.129212982	0.125778588	-1.027305073	0.304276835	0.554941773	NM_000484.3
105	Hck	22.85234665	0.715748114	0.566861997	1.262649671	0.206715109	0.435382264	NM_001172117.1
106	hFTL	9.645873524	0.126100592	0.597607041	0.211009214	0.832880081	0.926264686	NM_000146.3
107	hGRN	3.003641394	0.883222163	1.053945246	0.838015226	0.402022152	0.606072879	NM_002087.2
108	hITM2b	4.2048416	0.627325058	0.948778498	0.661192321	0.508488985	0.711884579	NM_021999.2
109	hLrrk2	19.68048002	-0.088672857	0.425779238	-0.208260172	0.835025828	0.926264686	NM_198578.2
110	HO-1	84.22307512	-0.089243538	0.303137012	-0.294400005	0.768452263	0.89366037	NM_010442.2
111	Hprt1	3011.15463	-0.192865373	0.10740727	-1.795645433	0.072550925	0.242330885	NM_013556.2
112	hSOD1	142407.46	-0.270558641	0.121960083	-2.218419625	0.026526234	0.123683035	NM_000454.4
113	Hspb1	317.5033807	-0.226789383	0.207756556	-1.0916112	0.275004031	0.532121621	NM_013560.2
114	Hspb2	71.68351457	-0.590592396	0.305549726	-1.932884719	0.053250401	0.20116818	NM_024441.2
115	hTDP43	4.7269634	-0.32047895	0.836588714	-0.383078262	0.701661737	0.834419577	NM_007375.3
116	IDE	331.7957386	0.185855364	0.216162022	0.859796565	0.389901193	0.602574571	NM_031156.2
117	IFIH1	62.59499796	0.511005674	0.3169547	1.612235675	0.106910665	0.318059228	NM_001164477.1
118	IFN-gamma	34.18428356	0.201709546	0.314840333	0.64067251	0.521735478	0.717127066	NM_008337.3
119	IGF1	66.22956731	-0.302123183	0.418994082	-0.721067901	0.470867739	0.67509953	NM_001111274.1
120	IGFBP2	285.4946982	-0.583095645	0.249551738	-2.336572162	0.019461441	0.094527001	NM_008342.3
121	IGFBP3	234.212813	-0.610109658	0.244000976	-2.50043941	0.012403935	0.065603033	NM_008343.2
122	IGFBP4	382.4101308	-0.359179687	0.19749098	-1.81871439	0.068955015	0.234447051	NM_010517.3
123	IGFBP5	199.3597806	-0.343756017	0.301170334	-1.141400655	0.253703232	0.503178077	NM_010518.2
124	IL-10	2004.206112	10.70682183	0.986377727	10.85468734	1.89E-27	1.50E-25	NM_010548.1
125	IL-1b	29.36938877	1.823526677	0.51769138	3.522420398	0.000427625	0.003769439	NM_008361.3
126	IL-4	6.418980022	-0.387966322	0.721445673	-0.537762351	0.590741127	0.747485331	NM_021283.2
127	IL-6	17.23951367	2.917810988	0.917807144	3.179111218	0.001477274	0.010654279	NM_031168.1
128	Il10rb	190.3887088	0.077231534	0.162283033	0.475906398	0.634141064	0.786070694	NM_008349.5
129	Il12a	5.456862028	-0.404066734	0.747895347	-0.540271758	0.589009632	0.747485331	NM_008351.2
130	Il1a	9.014357529	0.282525125	0.626915491	0.450659026	0.652235318	0.798156843	NM_010554.4
131	Il1r1	72.10115563	-0.213280709	0.220420005	-0.967610487	0.333238938	0.566506194	NM_001123382.1
132	Il1rn	16.14253541	1.507109205	0.700104273	2.152692481	0.031342855	0.138140731	NM_031167.5
133	Il6ra	40.98353125	-0.157963401	0.296004737	-0.533651598	0.593582603	0.747485331	NM_010559.2
134	Il7	17.84636372	-0.409946157	0.45003801	-0.910914516	0.362340416	0.582682562	NM_008371.2

135	Il8rb	13.91011996	-1.64523688	0.87964343	-1.870345216	0.061435894	0.221541559	NM_009909.3
136	Itgb2/MAC-1	93.23649837	0.925474092	0.358620432	2.580650765	0.009861429	0.055881429	NM_008404.4
137	ITM2b	5586.73382	-0.020105867	0.068690426	-0.292702606	0.769749478	0.89366037	NM_008410.2
138	JAK3	5.375063014	-0.145486144	0.832205226	-0.174820032	0.861221048	0.940232153	NM_001190830.1
139	Limk1	482.0943809	-0.39512121	0.132436112	-2.983485412	0.002849856	0.018840716	NM_010717.2
140	Lrp1	900.7427626	-0.14526755	0.111346699	-1.304641739	0.192014869	0.419261823	NM_008512.2
141	Lrrk2	79.02689395	0.014144645	0.210083339	0.067328731	0.946320005	0.967731326	NM_025730.3
142	Ltbr	62.23137133	-0.003590937	0.285249073	-0.012588777	0.989955874	0.990545114	NM_010736.3
143	Ly86	639.6183313	1.43664323	0.211621608	6.788736022	1.13E-11	3.37E-10	NM_010745.2
144	Ly96	43.08047647	0.012585645	0.337819965	0.037255481	0.970281302	0.978504025	NM_001159711.1
145	Lysozyme M (Lyz2)	454.0167746	-0.602931475	0.412702084	-1.460936348	0.14403291	0.372606877	NM_017372.3
146	Maff	17.33136134	-0.254717983	0.453781159	-0.561323401	0.574577092	0.747485331	NM_010755.3
147	Mafk	35.9816885	-0.305331761	0.305699151	-0.998798197	0.31789246	0.55952837	NM_010757.2
148	Map2k4	1425.046386	-0.197326556	0.111083932	-1.776373518	0.075671373	0.246709408	NM_009157.4
149	Map2k6	56.0004215	-0.209963553	0.298688304	-0.70295204	0.482085638	0.687044203	NM_011943.2
150	Mapk8	490.470852	-0.298881482	0.113820194	-2.625909087	0.00864179	0.051418648	NM_016700.3
151	Mapt	382.1418255	-0.1911427	0.154651212	-1.235959924	0.216473461	0.444143825	NM_001038609m.1
152	Maptex1	10.48602398	-0.316444238	0.59672822	-0.530298766	0.595904802	0.747485331	NM_005910ex1.1
153	Maptex10	40.35247636	1.860755183	0.633429529	2.937588316	0.003307759	0.021276939	NM_005910ex10.1
154	Maptex9_11	72.62810261	0.066465222	0.27760912	0.239420166	0.810779795	0.918883767	NM_001038609ex9_11.1
155	MARCO	26.34607934	2.627108545	0.846492767	3.103521552	0.001912323	0.013386261	NM_010766.2
156	Masp2	7.144244426	-0.095874474	0.812123886	-0.118054	0.906024871	0.960247484	NM_010767.3
157	Mbl2	3.49472495	-1.004937666	1.060527635	-0.947582725	0.343341924	0.568043242	NM_010776.1
158	Mef2b	4.84848241	-0.926459562	0.998144581	-0.928181728	0.353313311	0.572031075	NM_008578.2
159	Mef2c	155.5094029	-0.061008797	0.203422393	-0.299911902	0.764244356	0.89366037	NM_001170537.1
160	Mertk	70.22898927	-0.159210506	0.254174287	-0.626383211	0.531063619	0.718142848	NM_008587.1
161	Mfn1	261.2357485	-0.267783603	0.161930594	-1.653693701	0.098189763	0.299604662	NM_024200.4
162	MMP11	25.80271997	0.042599793	0.387791505	0.10985231	0.912526505	0.960247484	NM_008606.2
163	MMP2	95.51245963	-0.872798372	0.312146101	-2.796121334	0.005171998	0.03239304	NM_008610.2
164	MMP3	4.634224081	-0.334107508	0.795267425	-0.420119695	0.674398016	0.810640039	NM_010809.1
165	MMP9	52.11905005	-0.455152093	0.517686642	-0.87920386	0.379290752	0.59782251	NM_013599.2
166	Mrc1	166.0972246	0.374515117	0.254860967	1.469487937	0.141700491	0.370601285	NM_008625.2
167	Ms4a4a	15.89770443	-0.4181875	0.55183166	-0.757817157	0.44856045	0.654953295	XM_003086124.1
168	Ms4a6a	91.90397713	1.715073779	0.21940471	7.816941477	5.41E-15	2.15E-13	NM_026835.2
169	Msr1	33.9077417	0.704562638	0.347898669	2.025194976	0.042847347	0.169961144	NM_001113326.1
170	Myc	44.91013758	0.210184163	0.324033068	0.64865035	0.516564403	0.714780977	NM_010849.4
171	Myeloperoxidase	112.7696334	-0.644559249	0.679553681	-0.9485038	0.342873039	0.568043242	NM_010824.2
172	Myl2	16.35784745	-0.055342956	0.499745807	-0.110742213	0.911820771	0.960247484	NM_010861.3
173	Ncf1	106.0048408	0.415897648	0.327936816	1.26822494	0.204717633	0.435024971	NM_010876.3
174	Neprilysin	152.8413994	-0.284775234	0.204850245	-1.390163016	0.164479381	0.404230901	NM_008604.3
175	NLRP3	11.31601646	-0.211895687	0.763736096	-0.277446212	0.781437502	0.902825852	NM_145827.3
176	Nos2	3.116247036	0.787396076	1.165963409	0.675317999	0.499473751	0.703400903	NM_010927.3
177	NPY	209.2460409	-0.33885335	0.21696461	-1.561790886	0.11833726	0.327491486	NM_023456.2
178	P2ry12	266.3176122	-0.03195163	0.209139686	-0.152776502	0.878574529	0.941895215	NM_027571.3
179	P2RY6	37.65234101	0.822784706	0.323229837	2.545509761	0.010911831	0.060395717	NM_183168.2
180	Park9 (ATP13A2)	45.27334907	-0.287169694	0.282248154	-1.017436925	0.308945624	0.554941773	NM_001164366.1

181	Peripherin	445.6879727	-0.564032064	0.232186822	-2.429216522	0.015131493	0.076623303	NM_001163588.1
182	Pgk1	5301.183181	-0.160620041	0.116492935	-1.378796409	0.167957534	0.404230901	NM_008828.2
183	Pink1	1288.600065	-0.261921161	0.12761748	-2.05239252	0.040131531	0.164677661	NM_026880.2
184	Plasmin	5.509152618	-0.655757106	0.780002954	-0.840711053	0.400509827	0.606072879	NM_008877.3
185	PPP1R2	759.2491201	-0.269950218	0.141314046	-1.910285826	0.056096422	0.205399206	NM_025800.3
186	Prkcb1	660.8364374	-0.078875739	0.148582676	-0.530854212	0.595519809	0.747485331	NM_008855.2
187	protein phosphatase	3518.176001	-0.182253562	0.136105981	-1.339056234	0.180552367	0.410164508	NM_019411.4
188	PSD95	450.3996488	-0.178445516	0.129821198	-1.374548364	0.169271507	0.404230901	NM_001109752.1
189	PTAFR/PAFR	71.44067685	-0.003028397	0.255556226	-0.01185022	0.990545114	0.990545114	NM_001081211.1
190	PTGDS	10847.82796	-0.886635549	0.185128205	-4.789305604	1.67E-06	2.10E-05	NM_008963.2
191	PTGER1	10.50446251	-0.111240654	0.610062926	-0.182342918	0.855313619	0.938085905	NM_013641.2
192	PTGER4	15.01548642	0.143787465	0.55443188	0.259341986	0.795371387	0.914484976	NM_008965.1
193	PTGS2	24.15905155	0.216902875	0.40994227	0.529105903	0.596731987	0.747485331	NM_011198.3
194	Ptpn6	121.0241539	1.223642695	0.29246745	4.183859411	2.87E-05	0.00029657	NM_013545.2
195	PTPRC/CD45	149.5957074	1.137329783	0.303491859	3.747480366	0.00017862	0.001635058	NM_011210.3
196	S100A11	628.0088937	-0.159115137	0.231161156	-0.688329909	0.491245051	0.69593049	NM_016740.3
197	S100A8	2924.983337	-0.47669633	1.082546551	-0.440347188	NA	NA	NM_013650.2
198	S100A9	366.1141923	-0.63335096	1.064263909	-0.595107054	NA	NA	NM_009114.2
199	SERPINA1	18.10955516	-0.527442255	0.834383605	-0.63213401	0.527299313	0.717127066	NM_009243.3
200	SERPINA3	197.6117204	0.052920451	0.229677272	0.230412223	0.817771464	0.922415206	NM_009252.2
201	SerpinG1	149.7404413	-0.017751827	0.306766912	-0.057867475	0.953854191	0.970159391	NM_009776.3
202	Snap91	1264.625299	-0.144579314	0.123769666	-1.168132051	0.242753504	0.489621474	NM_013669.1
203	SOD1	5543.260045	-0.259512008	0.117363437	-2.211182755	0.027023184	0.123683035	NM_011434.1
204	Sox10	218.8138429	-0.312119037	0.19969325	-1.562992428	0.118054376	0.327491486	XM_128139.6
205	STAT3	553.9633604	0.124351185	0.108111051	1.150217148	0.250054445	0.50010889	NM_213659.2
206	STAT6	112.4091062	-0.252760178	0.246810892	-1.024104635	0.305785855	0.554941773	NM_009284.2
207	synaptophysin	1026.354232	-0.250867302	0.11226475	-2.234604389	0.025443332	0.121110262	NM_009305.2
208	Synuclein	139.4461099	-0.301887998	0.168568251	-1.790894767	0.073310184	0.242330885	NM_009221.2
209	TDP43	737.348434	-0.125852269	0.145238655	-0.866520481	0.3862048	0.600763022	NM_001003899.2
210	Tgfb1	98.33748139	0.237601815	0.263014381	0.903379557	0.366324486	0.585135756	NM_011577.1
211	Tgfb3	93.56036915	-0.023227343	0.280298551	-0.082866441	0.933957738	0.966955856	NM_009368.2
212	Tgfbr1	228.6382337	-0.1549595	0.143288912	-1.081447945	0.279497904	0.536455655	NM_009370.2
213	TIMP1	70.0877462	0.028669366	0.348591561	0.08224343	0.934453138	0.966955856	NM_001044384.1
214	Tlr1	22.05724811	0.828050652	0.411423892	2.012646006	0.044151883	0.172264725	NM_030682.1
215	Tlr2	22.35792179	0.39268674	0.413742897	0.949108112	0.342565629	0.568043242	NM_011905.2
216	Tlr4	49.12525534	0.217401112	0.295913801	0.734677166	0.46253613	0.671241457	NM_021297.2
217	Tlr6	25.78665244	0.750183582	0.443505711	1.691485732	0.09074407	0.280481672	NM_011604.3
218	Tlr7	21.19085089	0.094037497	0.467684601	0.201070331	0.840643581	0.926264686	NM_133211.3
219	TLr8	6.669783698	0.422770758	0.783402991	0.539659361	0.589431971	0.747485331	NM_133212.2
220	TLR9	24.06271357	0.602232118	0.40818083	1.475405197	0.140103639	0.370496289	NM_031178.2
221	TNFa	3.672929281	0.429330631	1.074601563	0.399525411	0.689506104	0.824635441	NM_013693.2
222	TNFRSF1A	142.8534306	-0.012718553	0.192789776	-0.065971097	0.947400836	0.967731326	NM_011609.4
223	TNFRSF1B	7.303172696	0.676805944	0.660869395	1.024114521	0.305781186	0.554941773	NM_011610.3
224	TNFSF11	6.579446478	0.382820659	0.786594769	0.486680912	0.626484475	0.780645576	NM_011613.3
225	TNFSF9	6.43442612	-0.765129466	0.725296006	-1.05492028	0.291461787	0.546203978	NM_009404.3
226	tPA	251.9357224	-0.285868256	0.167340657	-1.708301266	0.087580457	0.277921982	NM_008872.2

227	Transferrin	15023.96921	-0.216585029	0.258630603	-0.83743001	0.402350903	0.606072879	NM_133977.2
228	Trem-1	5.71442201	0.035374761	0.875473002	0.040406456	0.967769083	0.978504025	NM_021406.5
229	Trem-2	127.4788744	1.186409924	0.21718715	5.462615658	4.69E-08	8.59E-07	NM_031254.2
230	TREMl1	5.622078661	-0.541592001	0.852303071	-0.635445323	0.525138016	0.717127066	NM_027763.1
231	TREMl2	8.892712436	0.796637468	0.752149581	1.059147658	0.289532538	0.546203978	NM_001033405.2
232	TREMl4	9.110483546	-0.070198597	0.698087238	-0.100558488	0.919900951	0.960247484	NM_001033922.2
233	Tubb5	1268.900549	-0.211609119	0.099136436	-2.134524173	0.032799897	0.140104968	NM_011655.5
234	TyroBP/DAP12	799.9628722	0.710578782	0.204209911	3.479648851	0.000502071	0.004267607	NM_011662.2
235	Tyrosine hydroxylase	23.24362861	-0.41744283	0.37555663	-1.111530983	0.266339862	0.5238751	NM_009377.1
236	UbC	2443.363832	-0.272258088	0.108753389	-2.503444638	0.012299092	0.065603033	NM_019639.4
237	Ubiquilin1/2	174.8371541	-0.296525978	0.193494972	-1.532473816	0.125405554	0.339165022	NM_152234.2
238	VWF	219.6479321	-0.137829196	0.189917632	-0.725731438	0.468003425	0.675059486	NM_011708.3
239	ZBP1	113.7489311	2.174237256	0.464054873	4.685302071	2.80E-06	3.33E-05	NM_021394.2
240	zif268	72.61017483	-0.186270514	0.314927551	-0.591471002	0.554204879	0.736685574	NM_007913.5

Table S3: Differential Expression of Genes in AAV-IL10+AAV-M3 vs AAV-IL10 expressing SOD1-G93A mice

NanoString custom code-set readout. N=3 mice/group, basemean = mean normalized counts for all samples; lfcSE=standard error of fold change(log2), stat=Wald statistic; pvalue=Wald test p value; padj=Benjamini-Hochberg adjusted p value

	Row.names	baseMean	log2FoldChange	lfcSE	stat	pvalue	padj	accession$Accession
1	ABCA1	298.9425785	-0.009706331	0.131943993	-0.07356402	0.941357302	0.986852059	NM_013454.3
2	ACE	124.4289786	-0.316976274	0.201248351	-1.575050293	0.115244836	0.573962605	NM_009598.2
3	ADAM 10	847.2341408	-0.067614975	0.115924598	-0.583266849	0.559713675	0.89454015	NM_007399.3
4	ADAM 17/TACE	278.6022788	-0.137237299	0.145139635	-0.945553562	0.34437634	0.779519097	NM_001277266.1
5	Aif	261.4082923	-0.058561008	0.16478994	-0.355367613	0.722314208	0.950943492	NM_012019.2
6	ALOX5	45.90395839	-0.371539092	0.319978338	-1.161138265	0.245585676	0.729013077	NM_009662.2
7	ALOX5AP	63.62251462	-0.079803947	0.33784475	-0.23621485	0.813265957	0.973775106	NM_009663.1
8	alpha-syn	235.3203746	0.080364962	0.162272036	0.495248373	0.620424808	0.934754443	NM_000345.3
9	ApoE4	14220.98133	0.012337257	0.118675578	0.103957845	0.917202802	0.982272639	NM_009696.2
10	APP	2899.648533	-0.249047499	0.125115951	-1.990533552	0.046532192	0.410172654	NM_001198823.1
11	Aquaporin4	2176.041856	-0.001254851	0.220552449	-0.005689581	0.995460396	0.995460396	NM_009700.2
12	Arc/arg3_1	48.23670775	-0.245705406	0.284842105	-0.862602128	0.38835626	0.801546442	NM_018790.2
13	ARG1	18.20750062	0.733676138	0.447409648	1.639830838	0.101040344	0.546536408	NM_007482.3
14	Axi	48.75439169	0.030336611	0.262767596	0.115450348	0.908088173	0.979886443	NM_001159598.1
15	B2M	9222.488255	0.670182406	0.29046419	2.307280652	0.021039179	0.274739255	NM_009735.3
16	BACE1	554.1522959	-0.307770405	0.184939389	-1.664169041	0.096078653	0.531784173	NM_011792.4
17	BDNF	40.91004634	0.396000551	0.347200896	1.140551639	0.254056552	0.729013077	NM_007540.4
18	betaIII Tubulin	2474.199911	-0.062065216	0.145112424	-0.427704354	0.668866379	0.940078451	NM_023279.2
19	Bsn	110.5659627	-0.046718962	0.206698423	-0.226024765	0.821182159	0.973775106	NM_007567.2
20	C1qa	1074.476466	0.236332822	0.1878256	1.258256712	0.208298931	0.729013077	NM_007572.2
21	C1qb	1553.339675	0.613672456	0.235995243	2.600359426	0.009312616	0.221640272	NM_009777.2
22	C1r	151.5216614	0.218349058	0.316967378	0.688869182	0.490905595	0.862606166	NM_023143.3
23	C1s	55.15804772	0.371660252	0.371393742	1.000717594	0.316963359	0.760565691	NM_144938.2
24	c2	128.5216937	0.354165669	0.283510008	1.249217518	0.211585526	0.729013077	NM_013484.2
25	C3	238.5235094	-0.176827247	0.301087092	-0.587296007	0.557004922	0.89454015	NM_009778.2
26	C3ar1	110.949487	0.255867456	0.241577382	1.059153193	0.289530018	0.733065364	NM_009779.2
27	c4a	1069.665379	0.061509852	0.230673789	0.266652976	0.789736363	0.96218747	NM_011413.2
28	C5AR1	73.0658107	-0.210675282	0.278853639	-0.75550487	0.449946111	0.843206098	NM_007577.4
29	Calbindin	209.7170358	-0.002396362	0.172382087	-0.013901455	0.988908601	0.994717021	NM_009788.4
30	CARD9	44.61882706	-0.365349287	0.263027974	-1.389013045	0.164828783	0.700522329	NM_001037747.1
31	CASP1	83.33353754	0.51018053	0.297527173	1.714735916	0.086393656	0.505067365	NM_009807.2
32	CASP8	68.15248793	0.23087468	0.265051403	0.871056247	0.383723442	0.801546442	NM_009812.2
33	Catalase	890.8370753	-0.135340771	0.130761663	-1.035018732	0.300660163	0.741684529	NM_009804.2
34	Ccl11	76.66942305	0.583368147	0.472350071	1.23503347	0.216818048	0.729013077	NM_011330.3
35	Ccl17	23.39154374	-0.26449119	0.393011477	-0.672985918	0.50095625	0.863968025	NM_011332.3
36	Ccl19	53.26199701	-0.011801535	0.531394979	-0.022208594	0.982281562	0.994717021	NM_011888.2
37	CCL2	138.2659576	0.735187522	0.313079382	2.34824637	0.01886204	0.274739255	NM_011333.3
38	Ccl21b	9.107440464	-0.156966621	0.86588408	-0.181279024	0.856148569	0.974944304	NM_011335.2
39	Ccl3	13.637989	0.068920666	0.49632527	0.138861891	0.889559285	0.976440668	NM_011337.2
40	Ccl4	9.089102494	0.238614377	0.573095879	0.416360309	0.677146368	0.940078451	NM_013652.2
41	CCL5	193.505276	1.120252987	0.488195138	2.294682804	0.021751309	0.274739255	NM_013653.3

42	Ccl7	33.1338449	0.130497569	0.333046669	0.391829678	0.695184065	0.940078451	NM_013654.3
43	Ccl8	257.818888	0.594219213	0.188392737	3.154151395	0.001609656	0.063849671	NM_021443.2
44	CCND1	89.89735751	0.384375603	0.223832768	1.717244561	0.085934497	0.505067365	NM_007631.2
45	Ccr1	20.47612291	-0.189201623	0.432711658	-0.437246419	0.661932649	0.940078451	NM_009912.4
46	Ccr4	5.487981912	0.695432087	0.761630016	0.913083876	0.36119843	0.788671801	NM_009916.2
47	CD14	32.16972651	0.125994581	0.389430831	0.323535197	0.746289956	0.950943492	NM_009841.3
48	cd163	26.48868868	-0.186869889	0.533919403	-0.349996437	0.726341371	0.950943492	NM_053094.2
49	CD33	21.74844118	-0.023802532	0.370670793	-0.064214749	0.948799233	0.986852059	NM_001111058.1
50	CD36	55.26674877	-0.173696322	0.498183989	-0.348658981	0.727345343	0.950943492	NM_001159555.1
51	CD39/ENTPD1	132.6882683	0.314856431	0.201507308	1.562506263	0.118168772	0.573962605	NM_009848.3
52	Cd55	257.8240245	0.136226469	0.198674861	0.685675422	0.492917809	0.862606166	NM_010016.2
53	Cd68	103.3113707	0.493533158	0.232573988	2.12204796	0.033833711	0.350105355	NM_009853.1
54	Cebpd	12.00662518	-0.225677636	0.570558454	-0.395538151	0.692445771	0.940078451	NM_007679.4
55	Cfb	23.80206441	0.492240534	0.388837605	1.265928316	0.205538748	0.729013077	NM_008198.2
56	Cfl1	3175.093576	0.064497109	0.113281662	0.569351719	0.569117475	0.89454015	NM_007687.5
57	Chi3l3 (YM1)	84.04503679	-3.375921853	1.174214879	-2.875046054	0.004039686	0.137349314	NM_009892.2
58	Cltc	6518.049991	-0.061987099	0.117694501	-0.526677954	0.598417248	0.918860032	NM_001003908.1
59	Clu/ApoJ	9591.838654	-0.092451236	0.204588547	-0.451888621	0.651349222	0.940078451	NM_013492.2
60	Connexin47	419.360947	-0.206658111	0.387952988	-0.532688541	0.5942492	0.918385127	NM_080454.3
61	CR3/ITGAM	119.9596671	-0.069190373	0.255574059	-0.270725336	0.786602289	0.96218747	NM_001082960.1
62	CR4/ITGAX	4.648425355	0.207775656	0.828823334	0.250687508	0.802055719	0.968981021	NM_021334.2
63	Creb1	192.3398892	0.107594658	0.152231724	0.706782104	0.479701898	0.858413922	NM_001037726.1
64	CRH	17.5378998	0.397458265	0.46284966	0.858720011	0.390495007	0.801546442	NM_205769.1
65	CRHR1	20.01612365	-0.331647951	0.413003942	-0.803014008	0.421966638	0.829839457	NM_007762.4
66	Crp	11.72678113	-0.235139986	0.563980284	-0.416929443	0.676730017	0.940078451	NM_007768.4
67	Csf1	242.0925127	-0.260603157	0.152360839	-1.710433993	0.08718565	0.505067365	NM_001113530.1
68	Csf1R/cd115	231.5484565	-0.030529293	0.179081513	-0.170477079	0.864634959	0.976440668	NM_001037859.2
69	Ctsb	290.2324331	0.125735708	0.127601804	0.985375629	0.324439596	0.764521028	NM_007798.2
70	Ctsc	447.1204377	0.359601926	0.174284722	2.063301487	0.039083993	0.381604519	NM_009982.4
71	Ctsd	6353.2232	0.124959455	0.106237809	1.176223942	0.2395054	0.729013077	NM_009983.2
72	CX3CR1	280.7878462	0.033996655	0.246451366	0.13794468	0.890284138	0.976440668	NM_009987.3
73	Cxcl10	102.2733474	0.984746111	0.399603447	2.464308348	0.013727796	0.272267948	NM_021274.1
74	Cxcl5	16.38827824	-0.220712218	0.612911835	-0.36010435	0.7187691	0.950943492	NM_009141.2
75	Cxcl8	7.181298405	-0.435932047	0.762706989	-0.571559005	0.567620776	0.89454015	NM_011339.2
76	Cxcl9	173.1767982	1.540314767	0.54440352	2.829362251	0.004664087	0.138756598	NM_008599.4
77	Cxcr4	52.34754286	-0.462144726	0.464303113	-0.995351339	0.319565417	0.760565691	NM_009911.3
78	CXCR7	61.15461774	0.225849095	0.271101606	0.833079148	0.404800093	0.820444339	NM_007722.3
79	Dock2	18.01498932	0.135664347	0.505423972	0.268416922	0.788378416	0.96218747	NM_033374.3
80	Drp1	918.6054013	0.041407711	0.122780426	0.337250099	0.735928374	0.950943492	NM_001025947.1
81	ECE1	174.1161309	-0.338846721	0.176530019	-1.91948498	0.054922985	0.454847749	NM_199307.2
82	ECE2	441.4016006	-0.2554535	0.19040265	-1.341648872	0.179709872	0.712849159	NM_177942.1
83	eif2alpha	400.4314765	0.208692036	0.138757249	1.504008167	0.132579264	0.618703232	NM_026114.3
84	Fcer1g	109.2290692	0.219263793	0.232168759	0.94441558	0.344957323	0.779519097	NM_010185.4
85	FcgRI	122.8317285	0.320062325	0.236893252	1.351082491	0.176669009	0.712664818	NM_010186.5
86	FcgRIIb	482.0005001	0.295876286	0.154460369	1.915548228	0.055422625	0.454847749	NM_001077189.1

87	FcgRIII	116.9542107	0.389164364	0.212179454	1.834128408	0.066634935	0.48232452	NM_010188.5
88	FcgRIV	232.3686288	0.807673703	0.348437	2.317990632	0.02044983	0.274739255	NM_144559.1
89	Fcrls	67.83999876	-2.471134282	0.406350423	-6.081288809	1.19E-09	2.84E-07	NM_030707.3
90	FcRn	291.3638239	0.16773025	0.162462995	1.032421262	0.301874816	0.741684529	NM_010189.3
91	Flii	302.5001484	-0.235970015	0.130664359	-1.805924865	0.070930076	0.48232452	NM_022009.1
92	Fos	69.6340725	-0.193939271	0.278301703	-0.696866993	0.485886036	0.862606166	NM_010234.2
93	FTL	1378.703858	0.041786616	0.089833631	0.465155597	0.641820034	0.940078451	NM_010240.2
94	GAPDH	12836.04364	0.073273891	0.090710538	0.807777054	0.419218937	0.829839457	NM_001001303.1
95	GBA	194.6803465	-0.325051434	0.171721311	-1.892900955	0.058371042	0.463076936	NM_001077411.1
96	GFAP	3510.793856	-0.150634571	0.320959426	-0.469325899	0.638836705	0.940078451	NM_001131020.1
97	Gls	1000.139973	-0.106802495	0.113262193	-0.942966862	0.345697853	0.779519097	NM_001081081.2
98	GRIN2a	121.8486106	-0.150276115	0.207423785	-0.724488345	0.46876598	0.858202332	NM_008170.2
99	GRN	646.3594499	0.030009129	0.157250945	0.190835923	0.848654143	0.974944304	NM_008175.3
100	Gusb	142.2564398	-0.003586356	0.16522178	-0.021706315	0.982682226	0.994717021	NM_010368.1
101	H2-Eb1	144.443374	0.638811745	0.5361409	1.191499743	0.233457457	0.729013077	NM_010382.2
102	H2Ea	224.1681037	2.467959181	1.947890791	1.266990528	0.205158677	0.729013077	NM_010381.2
103	hApoE4	8.412064619	0.585050159	0.669736025	0.873553366	0.382361532	0.801546442	NM_000041.2
104	hAPP	4308.511156	-0.216483652	0.125658617	-1.722791927	0.084926159	0.505067365	NM_000484.3
105	Hck	22.85234665	-0.493907971	0.535792371	-0.921827181	0.356618727	0.788671801	NM_001172117.1
106	hFTL	9.645873524	-0.436794824	0.564822915	-0.773330565	0.439326771	0.829839457	NM_000146.3
107	hGRN	3.003641394	1.541380867	1.107785137	1.391407788	0.164101804	0.700522329	NM_002087.2
108	hITM2b	4.2048416	0.779493777	0.946558834	0.823502723	0.41022217	0.820444339	NM_021999.2
109	hLrrk2	19.68048002	-0.005015201	0.42287695	-0.011859716	0.990537537	0.994717021	NM_198578.2
110	HO-1	84.22307512	-0.019928399	0.302047911	-0.065977608	0.947395653	0.986852059	NM_010442.2
111	Hprt1	3011.15463	0.192498484	0.107565479	1.789593524	0.07351928	0.486044131	NM_013556.2
112	hSOD1	142407.46	0.131431897	0.121963226	1.077635456	0.281196473	0.733065364	NM_000454.4
113	Hspb1	317.5033807	-0.189431035	0.207231852	-0.914101925	0.360663291	0.788671801	NM_013560.2
114	Hspb2	71.68351457	0.058687218	0.31065636	0.188913621	0.850160518	0.974944304	NM_024441.2
115	hTDP43	4.7269634	-0.234341101	0.830899113	-0.282033158	0.777918086	0.96218747	NM_007375.3
116	IDE	331.7957386	0.242043718	0.215699518	1.122133793	0.261805557	0.729013077	NM_031156.2
117	IFIH1	62.59499796	0.354028815	0.312140629	1.134196519	0.256712124	0.729013077	NM_001164477.1
118	IFN-gamma	34.18428356	0.070648343	0.307248065	0.229939098	0.818139094	0.973775106	NM_008337.3
119	IGF1	66.22956731	-0.451247477	0.416219384	-1.084157764	0.278294855	0.733065364	NM_001111274.1
120	IGFBP2	285.4946982	-0.142557391	0.250399121	-0.569320654	0.569138553	0.89454015	NM_008342.3
121	IGFBP3	234.212813	-0.2824038	0.244584757	-1.154625509	0.248243816	0.729013077	NM_008343.2
122	IGFBP4	382.4101308	-0.311656696	0.19707599	-1.581403676	0.113785768	0.573962605	NM_010517.3
123	IGFBP5	199.3597806	0.251270809	0.302898066	0.829555674	0.406790047	0.820444339	NM_010518.2
124	IL-10	2004.206112	3.319284226	0.859769828	3.860666096	0.000113078	0.009653244	NM_010548.1
125	IL-1b	29.36938877	0.228475925	0.470562931	0.485537449	0.627295155	0.938970106	NM_008361.3
126	IL-4	6.418980022	0.104484124	0.737737276	0.141627822	0.887373989	0.976440668	NM_021283.2
127	IL-6	17.23951367	3.285513011	0.928152877	3.539840356	0.000400369	0.023821964	NM_031168.1
128	Il10rb	190.3887088	0.126771052	0.16118578	0.786490298	0.431580301	0.829839457	NM_008349.5
129	Il12a	5.456862028	-0.473477938	0.733791931	-0.64524822	0.518766342	0.875648152	NM_008351.2
130	Il1a	9.014357529	-0.04586679	0.603036005	-0.076059787	0.939371533	0.986852059	NM_010554.4
131	Il1r1	72.10115563	0.162986532	0.223460561	0.729374937	0.465772345	0.858202332	NM_001123382.1

132	Il1rn	16.14253541	0.91856484	0.674664038	1.361514455	0.173351169	0.711337555	NM_031167.5
133	Il6ra	40.98353125	-0.065782348	0.294208815	-0.223590677	0.823075819	0.973775106	NM_010559.2
134	Il7	17.84636372	-0.060305504	0.454995607	-0.13254085	0.894556515	0.97662592	NM_008371.2
135	Il8rb	13.91011996	-2.080995197	0.869876296	-2.392288658	0.016743668	0.274739255	NM_009909.3
136	Itgb2/MAC-1	93.23649837	-0.12639469	0.347513433	-0.363711667	0.71607333	0.950943492	NM_008404.4
137	ITM2b	5586.73382	0.00262137	0.068586641	0.038219829	0.969512411	0.99468366	NM_008410.2
138	JAK3	5.375063014	0.132316019	0.837581444	0.157973914	0.874477355	0.976440668	NM_001190830.1
139	Limk1	482.0943809	-0.280589422	0.132190484	-2.122614383	0.033786179	0.350105355	NM_010717.2
140	Lrp1	900.7427626	-0.188367834	0.110804127	-1.700007393	0.089129535	0.505067365	NM_008512.2
141	Lrrk2	79.02689395	0.038359242	0.207689978	0.184694717	0.85346849	0.974944304	NM_025730.3
142	Ltbr	62.23137133	0.147896123	0.2848172	0.519266824	0.603574685	0.920838302	NM_010736.3
143	Ly86	639.6183313	0.548423107	0.208847612	2.625948661	0.008640785	0.221640272	NM_010745.2
144	Ly96	43.08047647	-0.186579602	0.331329974	-0.563123221	0.57335098	0.89454015	NM_001159711.1
145	Lysozyme M (Lyz2)	454.0167746	-0.320354137	0.412824483	-0.776005664	0.437745642	0.829839457	NM_017372.3
146	Maff	17.33136134	0.54277021	0.474266548	1.14444127	0.252440681	0.729013077	NM_010755.3
147	Mafk	35.9816885	-0.346979148	0.300929077	-1.153026327	0.248899579	0.729013077	NM_010757.2
148	Map2k4	1425.046386	-0.041820252	0.111043131	-0.376612691	0.706461455	0.949931222	NM_009157.4
149	Map2k6	56.0004215	0.175293408	0.301719575	0.580981225	0.561253108	0.89454015	NM_011943.2
150	Mapk8	490.470852	0.081897476	0.114692265	0.714062767	0.475188368	0.858413922	NM_016700.3
151	Mapt	382.1418255	-0.185822825	0.153932485	-1.207170949	0.227366316	0.729013077	NM_001038609m.1
152	Maptex1	10.48602398	-0.753008767	0.573754188	-1.312424002	0.189377113	0.715424648	NM_005910ex1.1
153	Maptex10	40.35247636	2.468857681	0.642477034	3.842717402	0.00012168	0.009653244	NM_005910ex10.1
154	Maptex9_11	72.62810261	0.307356716	0.278378358	1.10409702	0.269551054	0.729013077	NM_001038609ex9_11.1
155	MARCO	26.34607934	0.779465824	0.798405336	0.976278325	0.328926558	0.767495302	NM_010766.2
156	Masp2	7.144244426	-0.82042	0.774248159	-1.059634422	0.289310947	0.733065364	NM_010767.3
157	Mbl2	3.49472495	-1.043845121	1.048046823	-0.995990922	0.319254556	0.760565691	NM_010776.1
158	Mef2b	4.84848241	-1.35694086	0.971570382	-1.396647001	0.162519749	0.700522329	NM_008578.2
159	Mef2c	155.5094029	-0.038062516	0.202177445	-0.188262921	0.85067055	0.974944304	NM_001170537.1
160	Mertk	70.22898927	0.080857116	0.254986914	0.317103002	0.751165447	0.950943492	NM_008587.1
161	Mfn1	261.2357485	-0.143049104	0.161607813	-0.885162053	0.376069235	0.801546442	NM_024200.4
162	MMP11	25.80271997	-0.055153198	0.380754121	-0.144852531	0.884827306	0.976440668	NM_008606.2
163	MMP2	95.51245963	0.098121149	0.318509957	0.30806305	0.758034357	0.954561782	NM_008610.2
164	MMP3	4.634224081	0.538680317	0.843744223	0.638440302	0.523187103	0.87689106	NM_010809.1
165	MMP9	52.11905005	-0.834016435	0.513040604	-1.625634362	0.104027461	0.550189682	NM_013599.2
166	Mrc1	166.0972246	0.080805075	0.251852574	0.320842762	0.748329552	0.950943492	NM_008625.2
167	Ms4a4a	15.89770443	0.07106624	0.560301027	0.126835819	0.899070345	0.977071882	XM_003086124.1
168	Ms4a6a	91.90397713	0.342549002	0.18850829	1.817156174	0.069193203	0.48232452	NM_026835.2
169	Msr1	33.9077417	0.369859337	0.334962609	1.104180968	0.269514644	0.729013077	NM_001113326.1
170	Myc	44.91013758	-0.088897638	0.31548473	-0.281781113	0.778111354	0.96218747	NM_010849.4
171	Myeloperoxidase	112.7696334	-2.127973882	0.674458031	-3.155087173	0.001604502	0.063849671	NM_010824.2
172	Myl2	16.35784745	-0.129504755	0.492022972	-0.263208757	0.792389681	0.96218747	NM_010861.3
173	Ncf1	106.0048408	-0.183647059	0.321956128	-0.570410199	0.568399514	0.89454015	NM_010876.3
174	Neprilysin	152.8413994	0.232176921	0.207527572	1.118776265	0.2632356	0.729013077	NM_008604.3
175	NLRP3	11.31601646	-0.048305902	0.763224959	-0.063291827	0.949534124	0.986852059	NM_145827.3
176	Nos2	3.116247036	0.242459206	1.10602619	0.219216514	0.826481392	0.973775106	NM_010927.3

177	NPY	209.2460409	0.096414894	0.218331199	0.441599251	0.658779226	0.940078451	NM_023456.2
178	P2ry12	266.3176122	0.482897901	0.210731949	2.291526764	0.021932966	0.274739255	NM_027571.3
179	P2RY6	37.65234101	0.126561196	0.30147764	0.419802927	0.674629427	0.940078451	NM_183168.2
180	Park9 (ATP13A2)	45.27334907	-0.205432501	0.280391441	-0.732663236	0.463763852	0.858202332	NM_001164366.1
181	Peripherin	445.6879727	-0.09576712	0.232837721	-0.411304145	0.680849531	0.940078451	NM_001163588.1
182	Pgk1	5301.183181	0.100025146	0.116524625	0.858403502	0.390669694	0.801546442	NM_008828.2
183	Pink1	1288.600065	0.163401448	0.12798604	1.276713059	0.201703569	0.729013077	NM_026880.2
184	Plasmin	5.509152618	-0.233403173	0.792749205	-0.294422463	0.768435105	0.96218747	NM_008877.3
185	PPP1R2	759.2491201	-0.004070949	0.141504168	-0.02876911	0.977048737	0.994717021	NM_025800.3
186	Prkcb1	660.8364374	-0.097890826	0.148079725	-0.66106839	0.508568456	0.870786277	NM_008855.2
187	protein phosphatase	3518.176001	-0.013606417	0.136099096	-0.099974337	0.9203647	0.982272639	NM_019411.4
188	PSD95	450.3996488	-0.235034464	0.128842659	-1.824197556	0.068122211	0.48232452	NM_001109752.1
189	PTAFR/PAFR	71.44067685	0.08534694	0.254265144	0.335661188	0.737126377	0.950943492	NM_001081211.1
190	PTGDS	10847.82796	0.281799704	0.185252528	1.521165226	0.128218379	0.610319486	NM_008963.2
191	PTGER1	10.50446251	-0.333483079	0.59485456	-0.560612798	0.575061525	0.89454015	NM_013641.2
192	PTGER4	15.01548642	0.026484525	0.545219936	0.048575856	0.961257311	0.99468366	NM_008965.1
193	PTGS2	24.15905155	0.015233993	0.39983772	0.038100439	0.969607601	0.99468366	NM_011198.3
194	Ptpn6	121.0241539	0.12699793	0.280599409	0.452595145	0.650840295	0.940078451	NM_013545.2
195	PTPRC/CD45	149.5957074	0.230142219	0.295813022	0.777998942	0.436569637	0.829839457	NM_011210.3
196	S100A11	628.0088937	-0.361713656	0.230531033	-1.569045393	0.116637362	0.573962605	NM_016740.3
197	S100A8	2924.983337	-1.759272458	1.082440642	-1.625283078	NA	NA	NM_013650.2
198	S100A9	366.1141923	-1.810831414	1.06348053	-1.702740542	NA	NA	NM_009114.2
199	SERPINA1	18.10955516	-1.780862188	0.812323935	-2.192305448	0.028357459	0.321384531	NM_009243.3
200	SERPINA3	197.6117204	0.519327703	0.231370019	2.24457648	0.024795334	0.295064476	NM_009252.2
201	SerpinG1	149.7404413	0.357126182	0.307865421	1.160007451	0.246045772	0.729013077	NM_009776.3
202	Snap91	1264.625299	-0.162345185	0.123460466	-1.31495684	0.188524408	0.715424648	NM_013669.1
203	SOD1	5543.260045	0.174251526	0.117460105	1.483495412	0.137942835	0.631353743	NM_011434.1
204	Sox10	218.8138429	-0.34408623	0.198548019	-1.73301266	0.083093419	0.505067365	XM_128139.6
205	STAT3	553.9633604	-0.140405322	0.106222083	-1.321809155	0.186231709	0.715424648	NM_213659.2
206	STAT6	112.4091062	-0.027974528	0.24718694	-0.113171546	0.909894554	0.979886443	NM_009284.2
207	synaptophysin	1026.354232	-0.133673335	0.112129872	-1.192129561	0.233210447	0.729013077	NM_009305.2
208	Synuclein	139.4461099	-0.158107035	0.168186141	-0.940071719	0.347180774	0.779519097	NM_009221.2
209	TDP43	737.348434	0.088265301	0.145313525	0.607412839	0.54357697	0.89454015	NM_001003899.2
210	Tgfb1	98.33748139	-0.183090415	0.257005304	-0.712399365	0.476217509	0.858413922	NM_011577.1
211	Tgfb3	93.56036915	0.312184553	0.281817464	1.10775446	0.267967872	0.729013077	NM_009368.2
212	Tgfbr1	228.6382337	0.170636113	0.144389921	1.181773017	0.237295777	0.729013077	NM_009370.2
213	TIMP1	70.0877462	0.887558053	0.356569347	2.489159709	0.012804543	0.272267948	NM_001044384.1
214	Tlr1	22.05724811	0.153487996	0.382843214	0.400916068	0.68848192	0.940078451	NM_030682.1
215	Tlr2	22.35792179	0.446278308	0.409889718	1.088776539	0.27625244	0.733065364	NM_011905.2
216	Tlr4	49.12525534	0.336924709	0.294770874	1.143005428	0.253036337	0.729013077	NM_021297.2
217	Tlr6	25.78665244	-0.031451019	0.418880369	-0.075083535	0.940148248	0.986852059	NM_011604.3
218	Tlr7	21.19085089	-0.145939051	0.456949551	-0.319376726	0.749440858	0.950943492	NM_133211.3
219	TLr8	6.669783698	0.3819656	0.771539805	0.495069208	0.620551269	0.934754443	NM_133212.2
220	TLR9	24.06271357	-0.078768061	0.383326333	-0.205485652	0.837192707	0.974944304	NM_031178.2
221	TNFa	3.672929281	0.443789995	1.062709681	0.417602289	0.676237923	0.940078451	NM_013693.2

222	TNFRSF1A	142.8534306	-0.0765086	0.190571958	-0.401468298	0.688075374	0.940078451	NM_011609.4
223	TNFRSF1B	7.303172696	0.246340301	0.623655437	0.394994233	0.692847144	0.940078451	NM_011610.3
224	TNFSF11	6.579446478	0.460898086	0.781053868	0.590097693	0.555125155	0.89454015	NM_011613.3
225	TNFSF9	6.43442612	-0.97429934	0.707214933	-1.377656628	0.168309328	0.702765265	NM_009404.3
226	tPA	251.9357224	-0.234820954	0.16660598	-1.409438927	0.15870542	0.700522329	NM_008872.2
227	Transferrin	15023.96921	-0.59685528	0.25859694	-2.308052372	0.020996224	0.274739255	NM_133977.2
228	Trem-1	5.71442201	-0.134547232	0.857939473	-0.156826019	0.875381966	0.976440668	NM_021406.5
229	Trem-2	127.4788744	0.209786749	0.203370503	1.031549538	0.30228319	0.741684529	NM_031254.2
230	TREMl1	5.622078661	-1.464718312	0.799803809	-1.831347007	0.067048766	0.48232452	NM_027763.1
231	TREMl2	8.892712436	-0.540533213	0.683776614	-0.790511408	0.429229163	0.829839457	NM_001033405.2
232	TREMl4	9.110483546	-0.712252836	0.666664685	-1.068382429	0.28534804	0.733065364	NM_001033922.2
233	Tubb5	1268.900549	-0.087250892	0.09902558	-0.881094478	0.378266686	0.801546442	NM_011655.5
234	TyroBP/DAP12	799.9628722	0.02898748	0.202706344	0.143002331	0.88628834	0.976440668	NM_011662.2
235	Tyrosine hydroxylase	23.24362861	0.082193776	0.38433267	0.213861018	0.83065544	0.973871895	NM_009377.1
236	UbC	2443.363832	-0.22158602	0.108636052	-2.039709795	0.041379239	0.381604519	NM_019639.4
237	Ubiquilin1/2	174.8371541	-0.125047069	0.193422289	-0.646497723	0.517957071	0.875648152	NM_152234.2
238	VWF	219.6479321	-0.128344304	0.188912114	-0.679386313	0.49689312	0.863215785	NM_011708.3
239	ZBP1	113.7489311	0.920169129	0.451811339	2.036622476	0.041687889	0.381604519	NM_021394.2
240	zif268	72.61017483	-0.347229518	0.311360104	-1.115202344	0.264763722	0.729013077	NM_007913.5
